# ECM alterations in Fndc3a (Fibronectin Domain Containing Protein 3A) deficient zebrafish cause temporal fin development and regeneration defects

**DOI:** 10.1038/s41598-019-50055-w

**Published:** 2019-09-16

**Authors:** Daniel Liedtke, Melanie Orth, Michelle Meissler, Sinje Geuer, Sabine Knaup, Isabell Köblitz, Eva Klopocki

**Affiliations:** 1 Institute of Human Genetics, Julius-Maximilians-University, Würzburg, Germany; 2 Institute for Medical Genetics and Human Genetics, Charité Universitätsmedizin Berlin, Berlin, Germany; 3 Center for Human Genetics, Bioscientia, Ingelheim, Germany; 4 Department of Cell and Developmental Biology, Julius-Maximilians-University, Würzburg, Germany

**Keywords:** Extracellular matrix, Limb development, Self-renewal

## Abstract

Fin development and regeneration are complex biological processes that are highly relevant in teleost fish. They share genetic factors, signaling pathways and cellular properties to coordinate formation of regularly shaped extremities. Especially correct tissue structure defined by extracellular matrix (ECM) formation is essential. Gene expression and protein localization studies demonstrated expression of *fndc3a* (*fibronectin domain containing protein* *3a*) in both developing and regenerating caudal fins of zebrafish (*Danio rerio*). We established a hypomorphic *fndc3a* mutant line (*fndc3a*^*wue1/wue1*^) via CRISPR/Cas9, exhibiting phenotypic malformations and changed gene expression patterns during early stages of median fin fold development. These developmental effects are mostly temporary, but result in a fraction of adults with permanent tail fin deformations. In addition, caudal fin regeneration in adult *fndc3a*^*wue1/wue1*^ mutants is hampered by interference with actinotrichia formation and epidermal cell organization. Investigation of the ECM implies that loss of epidermal tissue structure is a common cause for both of the observed defects. Our results thereby provide a molecular link between these developmental processes and foreshadow Fndc3a as a novel temporal regulator of epidermal cell properties during extremity development and regeneration in zebrafish.

## Introduction

A wide number of conserved genetic and structural features have been identified regulating fin development in ray finned fish species, like zebrafish (*Danio rerio*), and imply shared mechanisms throughout evolution^1^. The embryonic development of pectoral fins in fish species is assumed to resemble limb development in higher vertebrates, with common molecular signals arising from a structure called the apical ectodermal ridge (AER)^2,3^. Only recently differences between fin and limb AER have been reported and hint at a fin specific cellular process^3,4^. Moreover, there are also eminent developmental differences between paired fins (pectoral fins) and unpaired fins (caudal, anal and dorsal fins). All unpaired fins arise from a common developmental precursor structure, called the median fin fold (mff), which is exclusively found in teleosts^5^. An increasing number of molecular processes and distinct genes are still being identified by forward and reverse genetic screens in zebrafish, revealing a complex network of factors necessary for correct median fin fold development and function^6,7^. Already more than 30 years ago changes of epidermal cell shape and modulation of the extracellular matrix (ECM) have been described as one of the essential factors for correct median fin fold and caudal fin morphogenesis in zebrafish^8^. Recently, the Wnt signaling pathway has been shown crucial for regulation of epithelial cell morphology by modulating laminin levels and thereby orchestrating correct ECM patterning in growing fins^9^. These early cellular steps of caudal fin development are prerequisites for subsequent processes; i.e. mesoderm cell migration, cell differentiation, fin growth, development of cartilage and bone and the gradual resorption of the median fin fold during juvenile stages^10^. Particularly fin rays are essential structural elements formed by the assembly of actinotrichia and lepidotrichia at the basal membrane of epidermal cells^11,12^. While lepidotrichia are segmented and calcified bone rays, actinotrichia are non-calcified fibers with a characteristic brush-shaped structure. Actinotrichia fibers display a transverse striation which can be observed in several fish species via electron microscopy suggesting these fibrils to be hyperpolymerized collagen^13^. Two collagens, Col2a1 and Col1a1, as well as actinodin proteins, encoded by *and1, and2, and3* and *and4*, are essential for actinotrichia formation at the fin tips during fin development and regeneration^14–16^.

It is well accepted that a number of conserved molecular mechanisms are shared between extremity development during embryogenesis and fin regeneration in adult fish, a developmental process enabling complete replacement of lost tissues^17,18^. For both processes correct epidermal cell function, epithelial cell structure, and actinotrichia fiber assembly are essential to correctly build all skeletal and mesenchymal fin elements^14,19,20^. Structural factors, especially ECM proteins like integrins and laminins, have been implied in regulating Wnt signaling during regeneration and in correct assembly of the teleost fin^6,9,21^. Although a large number of involved “molecular players” have been described to date, not all factors necessary for correct ECM assembly in the regenerating caudal fin or correct median fin fold development and function have been elucidated yet.

*FNDC3A* protein has initially been described to be overexpressed in human odontoblasts^22^ and consists of up to nine fibronectin type III domains, which are a common feature of a large number of extracellular proteins acting by modulation of different signaling pathways^23,24^. Functional experiments in *Symplastic spermatids* (*sys*) knockout mice indicated that FNDC3A is essential for cell adhesion between spermatids and Sertoli cells, resulting in sterile males^25^. Further developmental functions of FNDC3A in vertebrates are still unknown and an association to extremity development in mammals has only been described recently by the Mouse Organogenesis Cell Atlas, showing expression of Fndc3a in epithelial cells of the limb AER^26^. The purpose of this study was to investigate potential functions of Fndc3a during vertebrate extremity deployment and regeneration in zebrafish (*Danio rerio*).

## Results

Phylogenetic and syntheny analyses showed that the *FNDC3A* gene is highly conserved throughout vertebrate evolution and orthologues are not duplicated in ray-finned fish species (data not shown). In the zebrafish genome *fndc3a* is located on chromosome 15 and encodes in 29 exons for two different transcripts that are highly similar, with corresponding proteins of 1247 and 1217aa that only differ in a 30aa stretch at the N-terminus (ENSEMBL Zv9: 3,066,162-3,114,443 reverse strand; ENSDARG00000067569; ZFIN ID: ZDB-GENE-030131-7015; GenBank: XM_021466300.1, XM_021466301). Zebrafish Fndc3a protein (UniProt: A0A140LGL5) consists of one transmembrane domain located at the C-terminus, 9 fibronectin type III domains and one signal peptide located at the N-terminus. Amino acid alignment resulted in an up to 57% amino acid identity with 95% coverage, indicating a high level of conservation between human and zebrafish proteins. Furthermore, two *fndc3a* paralogues can be identified in the zebrafish genome: *fndc3ba* (chromosome 2; ENSDARG00000078179; ZFIN ID: ZDB-GENE-070510-1) and *fndc3bb* (chromosome 24; ENSDARG00000062023; ZFIN ID: ZDB-GENE-070510-2). Both genes share highest sequence similarities with FNDC3B and form a distinct subgroup aside from FNDC3A gens. Amino acid alignment comparison of both zebrafish paralogous to human FNDC3B show for Fndc3ba up to 68%% amino acid identity by 98% coverage, while Fndc3bb shows up to 56% by 98% coverage. Both zebrafish proteins show typical FNDC3 protein domain structure, by displaying one transmembrane and 9 fibronectin type III domains. Syntheny analyses furthermore indicated the location of both genes within two distinct duplicated genomic regions on zebrafish chromosomes 2 and 24. Both regions share up to 8 additional duplicated genes flanking zebrafish *fndc3b* genes, which are also located within the human *FNDC3B* locus. All three gene family members have not been functionally investigated in zebrafish yet.

### Expression of *fndc3a* during early zebrafish development

**Figure 1:**
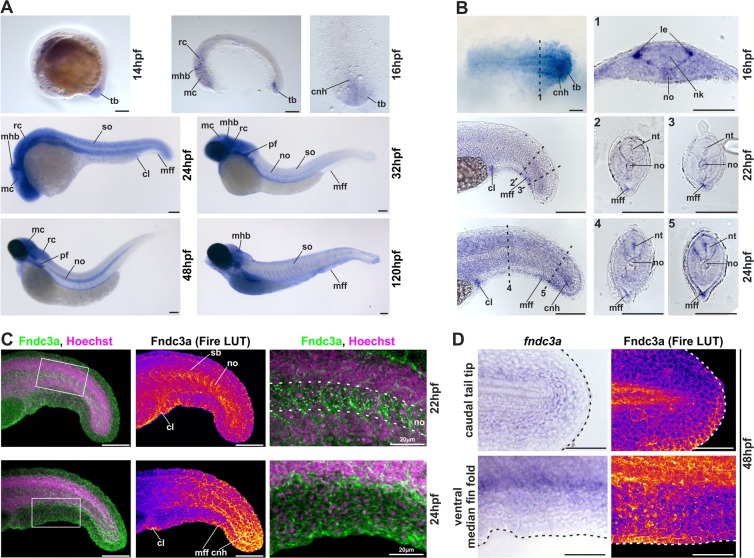
Localization of *fndc3a* RNA and protein during embryonic zebrafish development. (**A,B**) Expression of *fndc3a* mRNA was detected in the tail bud and the median fin fold from 14 hpf onwards. *fndc3a* is rather broadly expressed during embryogenesis, but was highly expressed in caudal and pectoral fins, somites, notochord cells and distinct brain regions. (**C,D**) Detection of Fndc3a protein via immunofluorescence indicated similar regional localization as *fndc3a* mRNA in 22–48 hpf embryos. Furthermore, it showed intracellular accumulation of Fndc3a at notochord cells, at somite boundaries and epidermal cells at this stage. *In-situ* stained embryos shown in (**A,B**) differ in proteinase K incubation and NBT/BCIP staining times to visualize weak *fndc3a* expression in different tissues and stages. Dashed lines in (**B**) indicate planes of the corresponding numbered sections 1–5, in (**C**) notochord boundary and in (**D**) fin fold border. Fire LUT in (**C,D**) shows pseudo-colored images of Fndc3a immunofluorescence and marks regions of high and low intensities (highest to lowest signal: yellow, red, blue, black). cnh: chordo neural hinge; cl: cloaca; le: lateral edge; mc: mesencephalon; mff: median fin fold; mhb: midbrain hindbrain boundary (marked with chevron); nk: neural keel; no: notochord; nt: neural tube; pf: pectoral fin; sb: somite boundary; so: somites; tb: tail bud; rc: rhombencephalon. Scale bars: 100 µm, except higher magnification in (**C**): 20 µm.

Earliest expression of *fndc3a* can be detected via RT-PCR and RNA-seq during blastula stages and indicate maternal transcripts of *fndc3a* (data not shown). To resolve the spatiotemporal expression of *fndc3a* during zebrafish development, we performed RNA *in-situ* hybridization experiments (Fig. 1). *fndc3a* transcripts were detected in a broad pattern and in number of different tissues, but showed cell type restricted expression within the tail bud region and the ventral median fin fold from 14 hpf onwards (hpf = hours post-fertilization; Fig. 1A,B; for visualization also of weak expression within the tailbud cells embryos shown in B are longer stained with NBT/BCIP). Expression in the tail bud region is changing during the next hours of development and could be detected apart from the median fin fold, in the cloaca, and in cells of the chordo neural hinge region (Fig. 1B). From 14 hpf onwards *fndc3a* was additionally present in distinct brain regions, the notochord, somites, pectoral fins and the caudal median fin fold, implying a rather broad and ubiquitous expression throughout zebrafish embryo development (Figs 1A and S4A).

Detection of Fndc3a protein localization was performed via immunofluorescence with a human FNDC3A antibody. Consistent with RNA *in-situ* hybridization this experiment showed similar regional localization of Fndc3a, e.g. in cloaca, the median fin fold region and the chordo neuronal hinge (22–24 hpf: Fig. 1C and 48 hpf: Fig. 1D). Specific Fndc3a protein localization could also be observed, as spots in notochord cells as well as in chevron shaped stripes between somite boundaries (higher magnification images in Fig. 1C). Moreover, this experiment clarified the cellular localization of Fndc3a at the cell membrane of epidermal cells during early stages of median fin fold development (higher magnification images Fig. 1C,D).

### Generation and phenotypic investigation of *fndc3a*^*wue1/wue1*^ mutants

**Figure 2:**
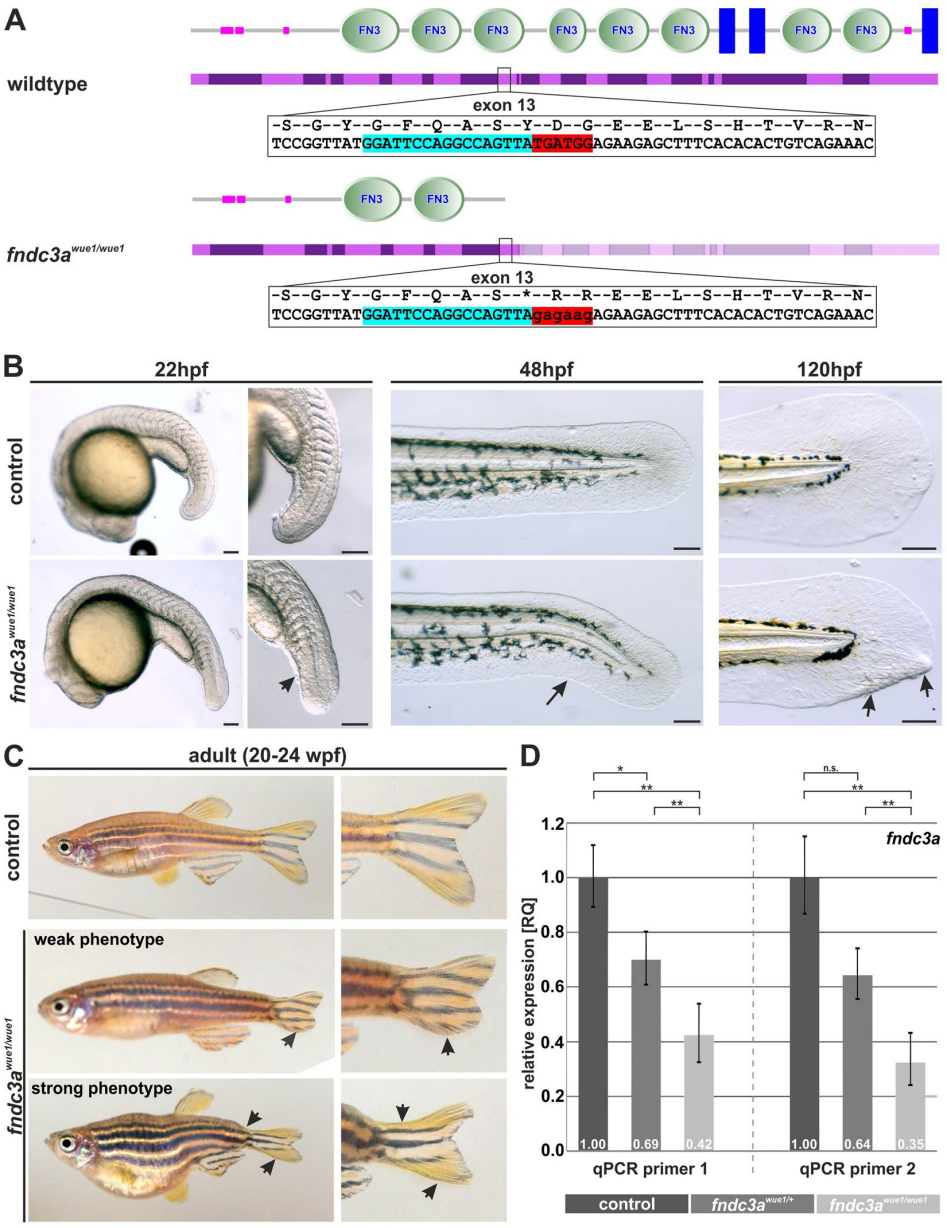
Generation and phenotype of *fndc3a*^*wue1/wue1*^ zebrafish mutants. (**A**) The CRISPR/Cas9 system was used to target exon 13 in the zebrafish *fndc3a* gene coding for the third fibronectin type III domain (nucleotides marked in light blue indicate sgRNA target sequence and in red the region of mutated sequence). (**B**) *fndc3a*^*wue1/wue1*^ mutants showed straightened tail buds (22 hpf; n = 19/40), kinked tails (48 hpf; n = 27/100), and caudal fin deformations (120 hpf; n = 9/41) during the first days of embryonic development. (**C**) A fraction of adult *fndc3a*^*wue1/wue1*^ mutants displayed weak (n = 15/71) to strong (n = 6/71) caudal fin phenotypes and tail malformations. (**D**) qPCR quantification of relative *fndc3a* expression levels in genotypic different groups of embryos indicated reduction of *fndc3a* transcripts in *fndc3a*^*wue1/*+^ and *fndc3a*^*wue1/wue1*^ (ΔΔCt calculation; significance levels of a 2-sided paired student t-test are given). Investigation of protein domains shown in A has been performed via the SMART database (Simple Modular Architecture Research Tool; http://smart.embl-heidelberg.de)^69^. Black arrows indicate developmental malformations. Scale bars for whole embryos: 250 µm; scale bars for tail magnifications: 100 µm.

To further investigate *fndc3a* function during zebrafish development, we established a mutant line via the CRISPR/Cas9 system^27,28^. sgRNAs were designed to target the evolutionary highly conserved third fibronectin III domain of Fndc3a (Fig. 2A), as incomplete genome information about the 5′end of *fndc3a* at the initiation of the project prevented the identification of a distinct start codon for sgRNA targeting. The established *fndc3a*^*wue1/wue1*^ mutant line has a 5 bp substitution leading to a premature Stop codon in exon 13 of *fndc3a* (Zv9: ENSDARE00000690608; *fndc3a*^*wue1*^ line; ZFIN ID: ZDB-ALT-170417-3; Fig. S1A). The alteration was validated by sequencing of genomic DNA and cDNA (Figs 2A and S1) and could be detected continuously in subsequent inbred generations (data not shown). The three most likely, computationally predicted off-site targets were sequenced and no off-site sequence alterations were detected in *fndc3a*^*wue1/*+^ *or fndc3a*^*wue1/wue1*^ mutants (Fig. S1B).

Visual inspection of *fndc3a*^*wue1/wue1*^ mutants showed a tail bud and median fin fold phenotype in homozygous embryos that could first be observed 22 hpf (Fig. 2B). Almost half of *fndc3a*^*wue1/wue1*^ embryos exhibited straightened tail buds and reduced ventral fin fold structures (22 hpf). During subsequent stages of development *fndc3a*^*wue1/wue1*^ mutant embryos displayed kinked tails (48 hpf) and caudal fin fold malformations (120 hpf). Due to the observed variability and the expectation of a stronger, more consistent phenotype after cellular stress, we further assessed the temperature sensitivity of the *fndc3a*^*wue1/wue1*^ phenotype^29,30^. The experiment revealed that raised temperatures indeed result in an increased number and severity of the caudal fin phenotype in mutants (Fig. S2).

Of adult *fndc3a*^*wue1/wue1*^ fish approximately 30% displayed alterations of the caudal fin shape and the posterior body part. The observed phenotype ranged from minor fin shape changes in the majority of affected fish, up to axis shortening and stronger caudal fin deformations (Fig. 2C). We did not observe changes in phenotype severity or appearance rates in subsequent, homozygous generations, excluding a potential stronger maternal zygotic effect in *fndc3a*^*wue1/wue1*^ or mitigation of the phenotype. Also, contrary to the mouse knock-out lines the *fndc3a*^*wue1/wue1*^ line did not show reduced fertility rates.

The observation of variable adult phenotypes, incomplete phenotypic penetrance and temperature sensitivity of the embryonic phenotype led us to the assumption that *fndc3a*^*wue1/wue1*^ mutants are hypomorphic. We tested this hypothesis by qPCR experiments, which revealed only partial loss of *fndc3a* transcripts due to nonsense mediated decay. We quantified *fndc3a* mRNA expression in three independent embryo groups with two primer pairs targeting different mRNA regions (Fig. 2D; primer sequences are given in Table S1). Relative to wildtype control fish *fndc3a* transcripts in *fndc3a*^*wue1/*+^ and *fndc3a*^*wue1/wue1*^ mutants were reduced to approximately 60% and 30% relative expression, respectively. Moreover, strong reduction of Fndc3a protein levels in mutants could be detected by immunofluorescence analyses on *fndc3a*^*wue1/wue1*^ mutants (Fig. S3).

To further validate the *fndc3a* phenotype we aimed to introduce a larger genomic deletion or multiple sequence alterations within the *fndc3a* locus by application of an additional sgRNA targeting exon 18. The corresponding gRNA was synthesized and co-injected with exon 13 sgRNA. The resulting transient phenotype indicated more severe tail fin malformations, i.e. strong caudal fin reduction and tail curling, when compared to *fndc3a*^*wue1/wue1*^ mutants (Fig. S4). This double *fndc3a* CRISPR phenotype could however not be maintained and next generation crossings did not result in a stable line. Nevertheless, it supported and confirmed our observations of the *fndc3a*^*wue1/wue1*^ mutant and pointed at a noticeable influence of Fndc3a on caudal fin development. No alterations in the development of pectoral fins could be detected in either *fndc3a*^*wue1/wue1*^ or the transient double CRISPR mutants (Figs S4 and S5).

### Reduction of Fndc3a function results in median fin fold defects

**Figure 3:**
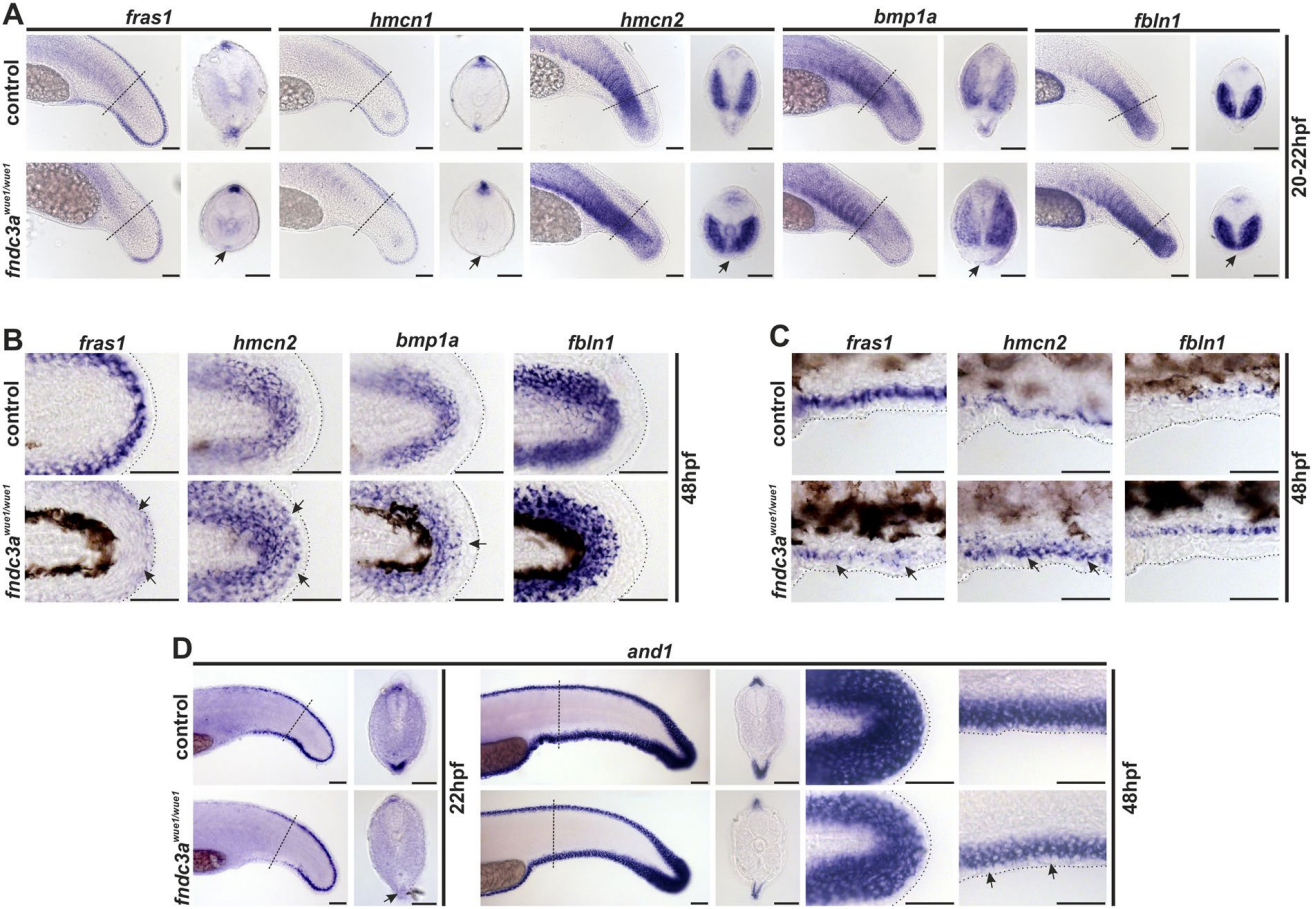
Normal development of the ventral median fin fold is altered in *fndc3a*^*wue1/wue1*^ mutants. (**A**) Investigation of *fras1*, *hmcn1*, *hmcn2*, *bmp1a*, and *fbln1* expression in the tail bud 20–22 hpf indicated loss of mesenchymal cells in the median fin fold in *fndc3a*^*wue1/wue1*^ mutants (arrows; *fras1*: 9/32; *hmcn1*: 11/26; *hmcn2*: 24/43; *bmp1a*: 22/45; *fbln1*: 16/32). (**B**) Expression of mesenchymal markers in posterior fins of 48 hpf old embryos was slightly changed following loss of *fndc3a* (*fras1*: 10/24; *hmcn2*: 12/25; *bmp1a*: 8/21; *fbln1*: 9/24). (**C**) Ventral fin folds in 48 hpf old embryos did not show loss of mesenchymal markers after *fndc3a*^*wue1/wue1*^ mutation. (**D**) Expression of *and1*, an essential marker for actinotrichia formation, in *fndc3a*^*wue1/wue1*^ in the ventral fin fold was partly lost in 22 hpf embryos (12/25), but recovered in 48 hpf old embryos to a reduced expression level (control n = 16; weaker expression in *fndc3a*^*wue1/wue1*^ embryos n = 10/18). Dashed lines in (**A,D**) indicate planes of shown sections. Dashed lines in (**B,C**) indicate fin boundaries. Scale bars: 50 µm

To elucidate the molecular causes underlying the early changes in caudal fin development of *fndc3a*^*wue1/wue1*^ mutants we analyzed expression patterns of different, well established markers, expressed in the median fin fold tissues (Table 1; Fig. 3).

**Table 1:** Summary of investigated median fin fold genes, their expression regions and corresponding references.

Gene	Region of expression	References
*fras1*	apical region of median fin fold	^6, 31, 32^
*hmcn1*	epithelial cells of apical fin fold	^6, 33^
*hmcn2*	fin fold epithelium and fin mesenchyme	^6, 33^
*bmp1a*	osteoblasts, fin mesenchyme cells, floor plate, hypochord cells	^34, 35^
*fbln1*	presomitic mesoderm cells, dorsal neural rod	^33, 36^
*and1*	essential factor for actinotrichia formation, median fin fold epithelium	^16^

All investigated early median fin fold genes were still expressed in the *fndc3a*^*wue1/wue1*^ embryos but showed altered patterns or intensity (Table 2). Affected regions in mutants correlated with the regions of investigated *fndc3a* expression. Observation of further mesodermal markers in *fndc3a*^*wue1/wue1*^ mutants 20–22 hpf additionally confirmed misplaced *myod* expression in ventral positions and indicated changes in chordo neural hinge cells by reduced *shha*, *ta(ntl)*, and *fgf8* expression (Fig. S6), hinting at a structural or steric effect on tail development after Fndc3a reduction. Moreover, gene expression analysis indicated that *fndc3a*^*wue1/wue1*^ embryos develop only temporal defects during early median fin fold and start to recover normal gene expression patterns in median fin fold cells 48 hpf. In summary, the *in-situ* experiments indicated, that most prominently apical cells expressing *fras1* and *hmcn1* located at the ventral median fin fold were influenced by reduced levels of Fndc3a during the first two days of zebrafish development.

**Table 2:** Summary of investigated alterations in median fin fold gene expression in *fndc3a*^*wue1/wue1*^ mutants at 20–22 hpf, at 48 hpf, after *fndc3a* Morpholino and *FNDC3A* RNA (rescue) injection at 20–22 hpf.

Gene	Alterations of gene expression in *fndc3a*^*wue1/wue1*^	
	20–22 hpf	48 hpf
*fras1* *hmcn1*	loss in ventral mff region between the cloaca and the tail bud tip	reduced expression
*hmcn2* *bmp1a* *fbln1*	loss of ventral mff structures posterior to proctodeum, convergence or even fusion of somites at ventral positions	similar to 20–24 hpf but slightly increased expression in cells of the posterior fin tip
*and1*	reduction in ventral mff region between the cloaca and the tail bud tip	recovered in fin folds compared to 20–22 hpf, but slightly weaker than wildtype
	**+** ***fndc3a*** **Morpholino**	**+** ***FNDC3A*** **RNA (rescue)**
*fras1* *hmcn1*	complete loss of ventral mff expression	regained ventral mff expression after RNA rescue
*hmcn2*	complete loss of ventral mff structures, ventrally shifted expression domain	regain of mff structures, expression domain not ventrally shifted
*bmp1a; fbln1; and1*	not analyzed	not analyzed

To validate specific effects on median fin fold development in *fndc3a*^*wue1/wue1*^ mutants Morpholino knockdown and rescue experiments were conducted (Figs S7 and S8). Phenotypic investigation of *fndc3a* morphants clarified that similar median fin fold malformations and comparable changes in median fin fold gene expression were induced in a dosage dependent manner in the first 48 h of development (Fig. S7B–D). Notably, injection of *fndc3a* Morpholino into *fndc3a*^*wue1/wue1*^ embryos resulted in an enhanced number of embryos showing tail phenotypes and supports the hypothesis of a hypomorphic mutation (Fig. S7B,C). Rescue and overexpression experiments were performed by injection of full-length human *FNDC3A* mRNA (Fig. S8). The tail phenotype and median fin fold gene expression in *fndc3a*^*wue1/wue1*^ mutants could be rescued after moderate RNA supplementation [25 ng/µl]. Injection of *FNDC3A* RNA at this concentration into wildtype embryos did not result in a raised number of tail malformations (Fig. S8B). Noteworthy, injection of higher RNA doses [50 ng/µl] resulted in a raised number of prominent tail deformations, especially in *fndc3a*^*wue1/wue1*^ mutants and to a smaller extent also in injected wildtype embryos. These experiments indicate a narrow threshold level for Fndc3a to fulfill its function in early median fin fold cells and support the observed *fndc3a*^*wue1/wue1*^ phenotype.

### *fndc3a*^*wue1/wue1*^ mutants show actinotrichia breakdown and defects in basal epidermal cells

**Figure 4:**
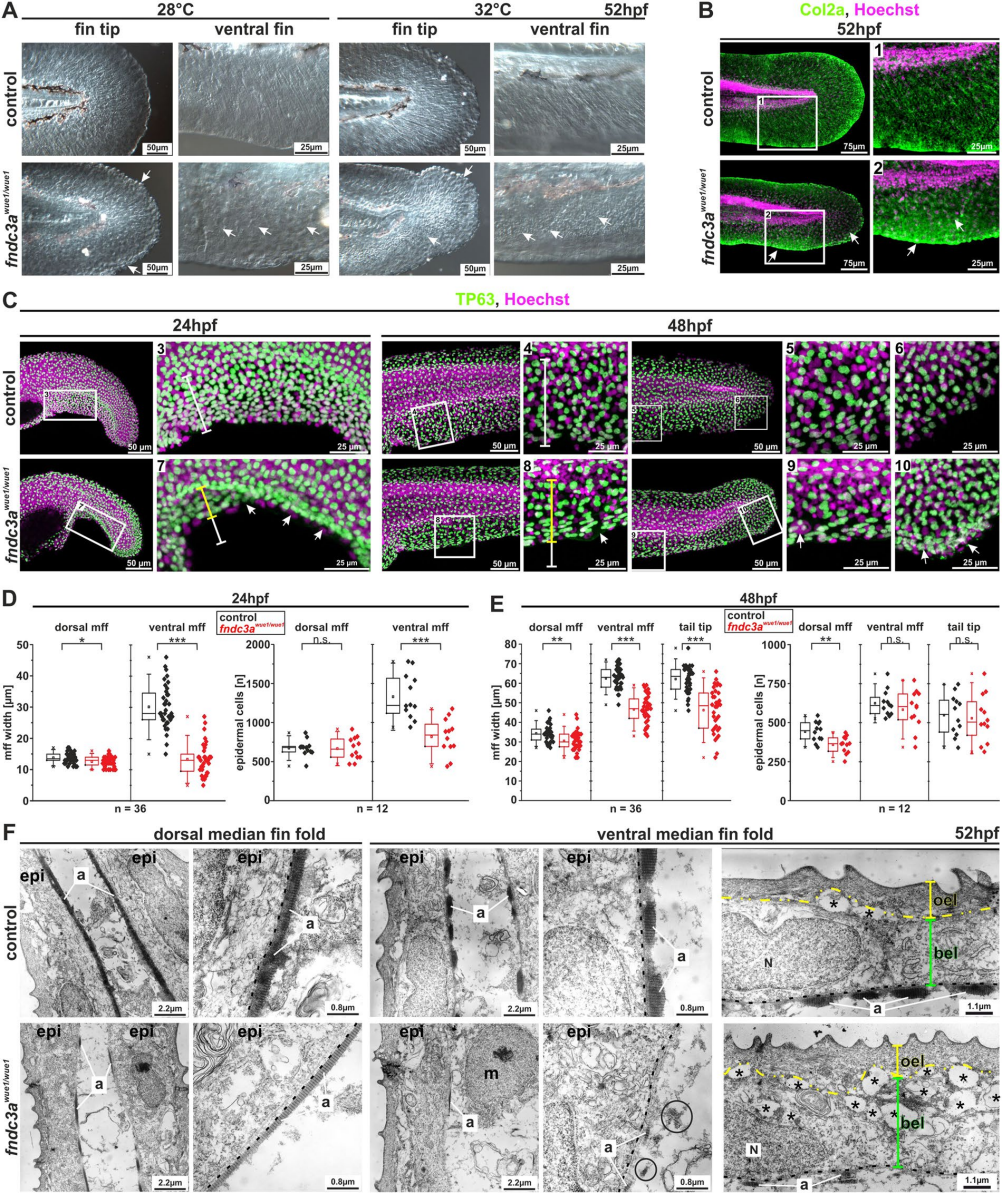
The *fndc3a*^*wue1/wue1*^ mutation results in structural defects in epidermal cells during fin development. Visualization of actinotrichia by either differential interference contrast microscopy (**A**) or immunofluorescence staining of Col2a (**B**) showed loss of mature actinotrichia in *fndc3a*^*wue1/wue1*^ mutants 52 hpf (control n = 0/17; *fndc3a*^*wue1/wue1*^ n = 19/26; white arrows indicate lost actinotrichia and Col2a accumulation in apical cells of the median fin fold). (**C**) Investigation of *fndc3a*^*wue1/wue1*^ mutants showed reduced median fin fold width and reduced TP63 positive epidermal cell number in the ventral median fin. (**D,E**) Quantification of median fin fold width and TP63 positive cells in 24 and 48 hpf embryos (Mann-Whitney U test; p < 0.05; two-tailed, significant U value changes are indicated with asterisks). (**F**) Ultrastructural analysis of dorsal and ventral fins of control and *fndc3a*^*wue1/wue1*^ mutants revealed breakdown of actinotrichia fibers and cellular malformations in cells of the basal epidermal layer in the ventral fin folds of 52 hpf old embryos (circles indicate misplaced fibers; dashed lines indicate the basal membrane; yellow brackets indicate outer epidermal cells and green brackets indicate basal epidermal cells; black asterisks indicate cavities). a: actinotrichia; bel: basal epidermal layer; epi: epidermis; mff: median fin fold; m: mesenchymal cell; N: nucleus; oel: outer epidermal layer.

Since our expression and knock-down observations implied a function of Fndc3a during early median fin fold establishment we subsequently investigated a potential link to epidermal cells and to associated structures, like actinotrichia fibers, in *fndc3a*^*wue1/wue1*^. Actinotrichia were visualized by either differential interference contrast (DIC) microscopy (Fig. 4A) or immunofluorescent staining of Col2a, a structural component of actinotrichia (Fig. 4B). This experiment revealed that actinotrichia fibers were still present in *fndc3a*^*wue1/wue1*^ mutants, but displayed obvious structural alterations and signs of breakdown in the caudal fin. While wildtype fish at 52 hpf showed radiant symmetrical arrangement of Col2a in the developing caudal fin, *fndc3a*^*wue1/wue1*^ embryos partly lacked these structures and depicted unstructured, crumbled collagen fibers in the fin mesenchyme. High levels of remaining Col2a in *fndc3a*^*wue1/wue1*^ mutants could be detected in apical cells at the fin border, which were visible as distinct cells in DIC microscopy.

Potential cellular reasons for actinotrichia breakdown and Col2 misallocation can be numerous. Based on the expression timing and localization of *fndc3a* RNA and Fndc3a protein during median fin fold development and the reduced function, we assumed that disturbance of epidermal cell structure might be causative for the observed phenotype and that the normal cellular organization within the developing mff is lost^8,16^. Indeed, investigation of TP63, an epithelial stem cell marker, expressed in all epidermal cells of the basal layer^37,38^ (Fig. 4C), showed a reduced number of epidermal cells in the ventral fin fold, lack of ventral fin fold structures and reduced median fin fold width at this position (arrows in Fig. 4C; quantification in Fig. 4D) in 24 hpf *fndc3a*^*wue1/wue1*^ mutants. At 48 hpf *fndc3a*^*wue1/wue1*^ mutants then developed outgrowing ventral median fin folds and regained ventral structures. Although these were still shorter in length they did not display a significant lower number of epidermal cells in comparison to control embryos (Fig. 4C; quantification in Fig. 4E), indicating cellular recovery of the median fin fold structure at this time. Additionally, *fndc3a*^*wue1/wue1*^ mutants displayed aggregation of epidermal cells at the fin fold border, which was not observed in control embryos (Fig. 4D10). Consistent with the phenotypic investigation, in pectoral fins no alteration in Col2a or TP63 immunofluorescence staining could be detected (Fig. S4C).

Transmission Electron Microscopy (TEM) further clarified the cellular consequences of Fndc3a reduction in the epidermal cell layer. While *fndc3a*^*wue1/wue1*^ mutant embryos showed only a slight reduction of actinotrichia in dorsal median fin folds (lower row Fig. 4F), an almost complete loss of actinotrichia in ventral median fin folds could be observed. Only small remains, showing the characteristic stratification, were still present at the basal membrane, in addition to misplaced collagen fibers not attached to the basal membrane (circles Fig. 4F). While mesenchymal cells, the outer and basal epidermal cell layer were present, the sub-cellular structure of epidermal cells was altered after *fndc3a* reduction. Cavities between the outer and basal epidermal layers (asterisks in Fig. 4F), as well as changes in the ECM surrounding these cells could be detected. This observation strongly implies that the disruption of actinotrichia fibers and the described effects on TP63 positive cells after Fndc3a reduction are due to cellular defects in the basal epidermal cell layer.

### Fin regeneration is also temporally influenced in *fndc3a*^*wue1/wue1*^ mutants

Besides their function during fin development, actinotrichia and epidermal cells possess essential roles during regeneration of adult caudal fins after amputation^16,39^. Similar to their role in fin development, Col2a and Col1a were identified to be necessary factors of actinotrichia formation also during regeneration^40^, implying comparable underlying cellular processes. We therefore hypothesized that reduction of Fndc3a function might also interfere with fin regeneration.

**Figure 5:**
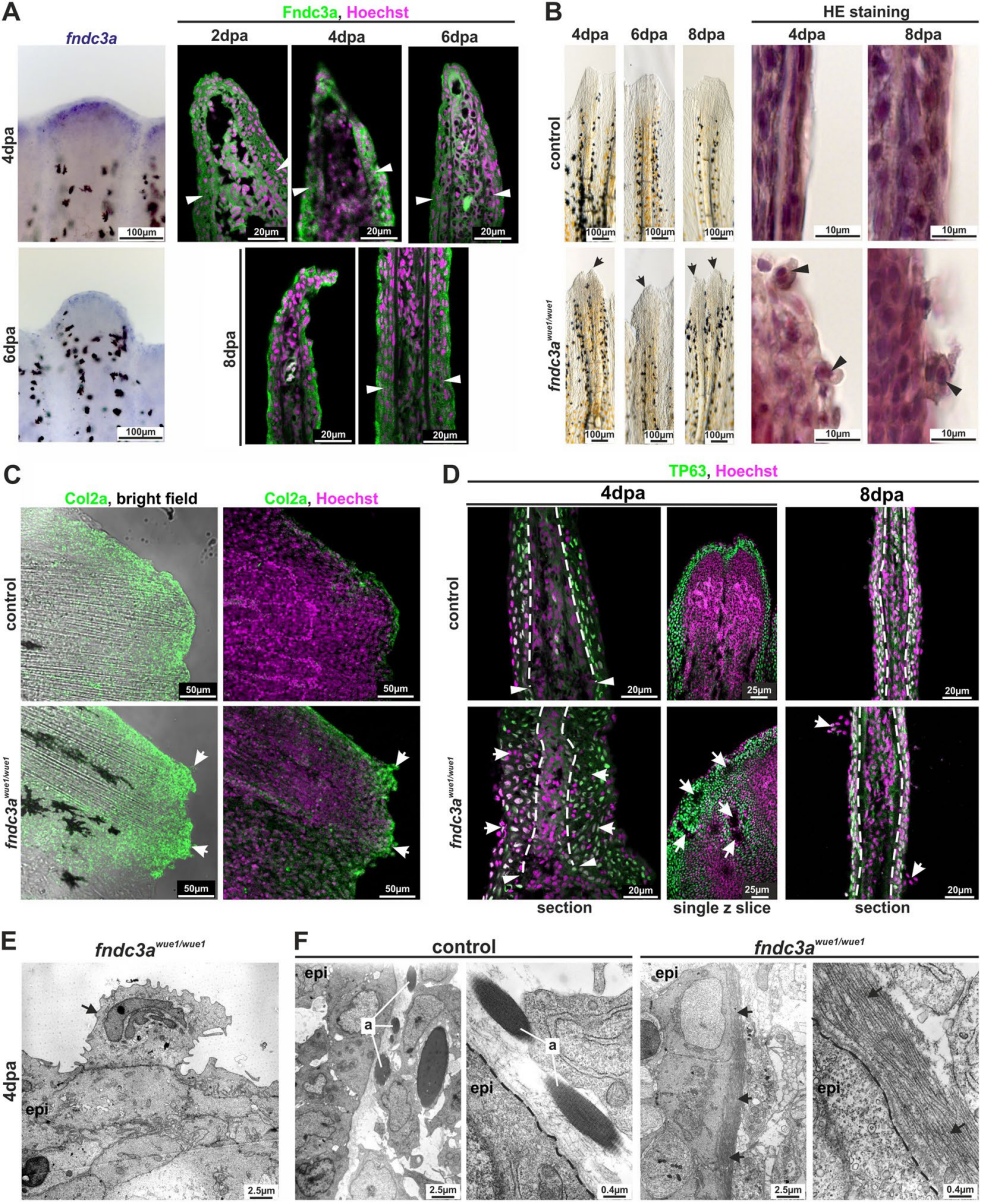
Interference with Fndc3a function during fin regeneration results in epidermal cells defects. (**A**) Expression of *fndc3a* and localization of Fndc3a in regenerates could be detected in epidermal cell layers. (**B**) Phenotypical and histological investigations of fin regeneration in *fndc3a*^*wue1/wue1*^ mutants indicated abnormal regeneration by showing unorganized fin borders at the regenerative front and aberrant epidermal cells (arrows; abnormal regenerate phenotypes, control: 3/19; *fndc3a*^*wue1/wue1*^: 13/19). (**C**) Col2a protein localization visualized via immunofluorescence on 8 dpa regenerates showed accumulation of collagen in abnormal cells of *fndc3a*^*wue1/wue1*^ mutants (white arrows; n = 6 for each group). (**D**) TP63 staining in regenerates of *fndc3a*^*wue1/wue1*^ mutants 4 and 8 dpa indicated disorganization of epidermal cells (arrows indicate epidermal thickening, cavities within the regenerate and abnormal cells; n = 4 for each group). (**E**) TEM analysis showed accumulation of electron dense material in the Golgi apparatus of abnormal cells attached to the epidermal layer (arrow). (**F**) TEM analyses of actinotrichia in fin regenerates 4 dpa revealed loss of these fibers located near the basal membrane of epidermal cells in *fndc3a* depleted fish (arrows). Arrowheads indicate amputation site. a: actinotrichia; epi: epidermis; dashed lines indicate basal membrane boundary.

Regeneration experiments on adult caudal fins confirmed *fndc3a* expression and localization of Fndc3a protein prominently in epidermal cells of regenerates between 2 and 8 days past amputation (dpa; Fig. 5A). Investigation of fin regeneration in *fndc3a*^*wue1/wue1*^ compared to wildtype fish led to two remarkable observations. First, regenerates looked opaque and disorganized, and aberrant cells attached to the epidermal layer were eminent between 4 dpa and 8 dpa in *fndc3a*^*wue1/wue1*^ mutants (arrows in Figs 5B and S9). Second, the apical fin borders of regenerates looked disorganized and clumped in *fndc3a*^*wue1/wue1*^ mutants (arrows in Fig. 5B).

Subsequent histological investigation via H&E and immunofluorescence staining of these regenerates confirmed that the aberrant cells were loosely attached cells that depicted high levels of Col2a at the regenerative front (arrows in Fig. 5B,C). TEM analysis further clarified that these cells were still attached to the epidermal cell layer and incorporated electron dense material in their Golgi apparatus and in intracellular vesicles (arrow in Fig. 5E). TP63 staining for epidermal cells in 4 to 8 dpa regenerates then showed interference with normal regenerate structure and disturbance of epidermal cell layers in *fndc3a*^*wue1/wue1*^ mutants (Fig. 5D). It also showed that actinotrichia fibers of *fndc3a*^*wue1/wue1*^ mutants only depicted loose collagen filaments adjacent to the membrane layer of epidermal cells and were lacking the prominent compact structure of bundles of stratified actinotrichia fibers in wildtype fish (Fig. 5F).

Furthermore, consistent with the hypomorphic *fndc3a*^*wue1/wue1*^ phenotype during fin development, the observed effects could be enhanced by keeping fish at a raised temperature of 32 °C during regeneration (Fig. S8). Although prominent cellular abnormalities were detected throughout the first days past amputation, a significant difference in regenerate length appeared only at 6 dpa. No significant differences in overall tail length growth could be detected between mutant and wildtype fish at all other investigated stages (Fig. S10). All regenerating fins grew to their former size after ~14 dpa (data not shown), but small changes in fin morphology, e.g. cooped fin rays and loss of interray tissues, could be observed in *fndc3a*^*wue1/wue1*^ mutants even 6 weeks past amputation (Fig. S9C). All this led to the assumption that a potential reason for the observed weak and transient effects is an altered epidermal cell organization in regenerates after reduced Fndc3a function, similar to the processes observed in the median fin fold during early development.

### *fndc3a*^*wue1/wue1*^ mutants display ECM alterations in median fin folds and in caudal fin regenerates

Correct assembly of actinotrichia and correct caudal fin morphology are greatly dependent on the extracellular composition and the cell shape of surrounding epidermal cells during development and regeneration. These cellular characteristics within the median fin fold determine correct signaling, e.g. by Wnt signals or cell behavior^18,41^. Fibronectin domain containing proteins like Fndc3a have been linked to functions during ECM assembly and maintenance^42^. Thus, we assumed that Fndc3a function might provoke cell shape or ECM alterations in epidermal cells.

**Figure 6:**
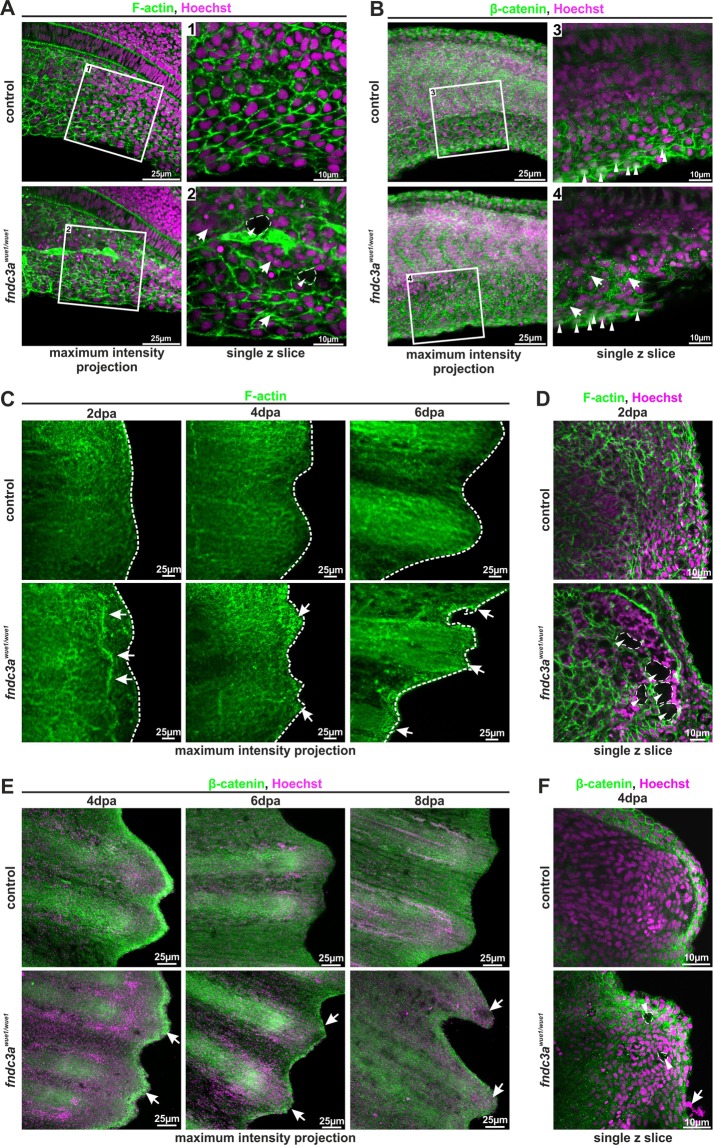
Correct ECM structure in the median fin fold and regenerating caudal fins is hampered in *fndc3a*^*wue1/wue1*^ mutants. (**A,B**) F-actin in the median fin fold was visualized by phalloidin staining and localization of β-catenin by immunofluorescence (n = 6 for each group, 22–24 hpf). Cellular organization of ventral median fin fold cells and ECM matrix was symmetrically structured in control embryos and showed nuclear localization of active Wnt signals in apical cells (white arrowheads in B). *fndc3a*^*wue1/wue1*^ mutants depicted cellular alterations and unstructured ECM assembly by showing irregular cell shapes (arrows in **A**), cavities within the fin fold (dashed lines in **A**) and speckled accumulation of β-catenin between cells (arrows in **B**). Nuclear localization of β-catenin in apical cells was maintained (arrowheads in **B**). (**C,D**) Fin regenerates of *fndc3a*^*wue1/wue1*^ mutants stained for F-actin showed regenerate abnormalities (arrows in **C**), irregular regenerate borders (dashed lines in **C**) and cellular cavities (dashed lines in **D**; n = 4 for each group). (**E**,**F**) Fin regenerates of *fndc3a*^*wue1/wue1*^ mutants stained for β-catenin depicted divergent ECM assembly (arrows in **E**), appearance of abnormal cells loosely attached to the regenerate (arrows in **F**) and cavities (dashed lines in **F**; n = 3 for each group). Images either show maximum intensity projections (30 to 40 single z-slices; z-distance: 1.5 µm) or a representative higher resolution single z slice.

To follow-up on this we analyzed F-actin and β-catenin localization in the median fin folds of wildtype and *fndc3a*^*wue1/wue1*^ mutants (Fig. 6). Cell boundaries of wildtype embryos 22 hpf displayed a dense, stereotypical assembly of epidermal cells in the ventral median fin fold (Fig. 6A,B). Reduction of Fndc3a resulted in clustering of these cells, altered cell shapes (arrows Fig. 6A) and appearance of cavities (dashed lines Fig. 6A). The cavities are zones within the fin folds showing no F-actin and no nuclear staining, suggesting cell free spaces. β-catenin in wildtype embryos 24 hpf displayed homogeneous membranous localization in cells of the ventral median fin fold and nuclear localization in epithelial cells at the fin border. In *fndc3a*^*wue1/wue1*^ mutants however uniform β-catenin localization was impaired and instead appeared as accumulations of protein in a speckled fashion within the cytoplasm (arrows in Fig. 6B). Nuclear localization of β-catenin in *fndc3a*^*wue1/wue1*^ mutants was still clearly detected at the apical fin border (arrowheads in Fig. 6B).

Comparison of wildtype and *fndc3a*^*wue1/wue1*^ mutants clarified that similar distinctive features were also observed during caudal fin regeneration. Irregular regenerate borders and cellular alterations of *fndc3a*^*wue1/wue1*^ mutants could be detected with both F-actin (dashed line and arrows Fig. 6C) and β-catenin staining (arrows in Fig. 6E). High resolution confocal microscopy of single z slices clarified, that the exhibited fragmented structures in F-actin staining in 2 dpa regenerates are cavities within the blastema which could also be seen with β-catenin staining 4 dpa (dashed lines Fig. 6D,F). Furthermore, it revealed β-catenin negative, detached cells outside of the regenerate (arrow in Fig. 6F). In summary, our observations in *fndc3a*^*wue1/wue1*^ mutants indicate a partial loss of cellular structure and adhesion within the blastema during caudal fin regeneration in adults as well as within the median fin fold during early embryonic development.

## Discussion

*fndc3a* expression in developing zebrafish embryos was first detected as maternal transcripts during blastula stages. During segmentation period stages (14 hpf) *fndc3a* expression was most prominent in the tail bud and later in apical cells of the ventral median fin fold, the pectoral fins, the notochord and in cells of the chordo neural hinge. Fndc3a protein localization showed comparable patterns and was subsequently detected in the cell membrane of ectoderm derived cells and in notochord cells. Spatiotemporally similar expression patterns in the zebrafish median fin fold have been described for other prominent genes also essential for vertebrate extremity development, e.g. *dlx5a* and *tp63*^38,43,44^. Comparison of zebrafish *fndc3a* during extremity development to mouse *Fndc3a* expression shows a partially similar pattern, i.e. early in the AER of the limb bud and later in interdigital regions of feet (Gene expression database; http://www.informatics.jax.org/expression.shtml), and was validated by single cell sequencing in the Mouse Organogenesis Cell Atlas (MOCA)^26^. Although expression of *Fndc3a* was detected in the AER of mice E10.5 and E11.5, mouse *Fndc3a* knockout result in male infertility, while defects in limb development were not described^25^.

The CRISPR/Cas9 generated *fndc3a*^*wue1*^ mutation in zebrafish, presented in our study, resulted in a premature Stop codon within exon 13. The mutation did not lead to complete nonsense mediated decay and full loss-of-function phenotype, as residual *fndc3a* mRNA expression could be detected and the observed phenotypes are rather weak and partly transient. Our data implies a potential residual expression level and only diminished function of Fndc3a in these mutants. Although tissue specific effects can also accrue when changed protein levels are interfering with signaling thresholds, signaling equilibriums or tissue structure. Morpholino knockdown and RNA overexpression experiments in wild type and in *fndc3a*^*wue1/wue1*^ mutants, respectively, displayed on the one hand a consistent phenotype of the mutant and further a partial rescue of the induced mutation. On the other hand, the experiments resulted also in an increased severity after Morpholino and RNA injection into *fndc3a*^*wue1/wue1*^, implying that the induced mutation does not abolish the function of the gene completely, but rather leads to a reduced function and has to be considered a hypomorphic mutation. RNA injections also showed, that at higher concentrations of FNDC3A RNA [50 ng/µl] changed fndc3a levels result in tail deformations. Interestingly, in mutants the phenotype penetrance was even enhanced at these higher concentrations. This observation hints to a narrow threshold level of functional Fndc3a at the mff to guarantee its normal development and indicates alternative or compensating cellular processes for mff development in the mutants. Our results cannot exclude a compensation mechanism for fin development and regeneration in *fndc3a*^*wue1/wue1*^ mutants, which despite of reduced Fndc3a function would be able to recover cellular functions and ultimately lead to a rather normally developed caudal fin during larval development and in adult fish. Compensation could either be facilitated by residing functional Fndc3a proteins, as indicated by remaining transcripts in mutants, by other fibronectin homologues, or by activation of known signaling networks during fin development and regeneration^18^. Especially tissues, like neuronal tissues, notochord and pectoral fins, in which *fndc3a* is strongly expressed but which display no defects in mutants should be investigated for compensatory mechanisms in close detail to reveal the involved factors for this process. Potential compensating factors might be determined by looking at other orthologues of the *fndc3* gene family, e.g. *fndc3ba* and *fndc3bb*, although expression patterns as well as function of these genes have not been investigated yet. Dorsophila *miles to go* mutants develop after complete depletion of the FNDC3 orthologue severe developmental defects, pupal lethality, neuromuscular junction defects and indicate a rather severe embryonic phenotype after complete loss of all FNDC3 functions^45^. Our experiments in zebrafish targeted one vertebrate FNDC3 orthologue and therefore might resemble only a fraction of the full FNDC3 loss-of-function phenotype.

A specific phenotype in the developing median fin fold of *fndc3a*^*wue1/wue1*^ mutants i.e. loss of ventral median fin fold cells, loss of gene expression domains in the ventral fin fold, loss of actinotrichia fibers and Col2a accumulation was observed. Most prominently a number of early markers in the ventral median fin fold were reduced or altered in *fndc3a*^*wue1/wue1*^ mutants, indicating the need of cellular Fndc3a function in this specific region and early phase of development. In contrast to more globally expressed genes linked to early caudal fin development and to actinotrichia deposition in zebrafish, e.g. *pinfin* mutants lacking Fras1 function or *nagel* mutants lacking Hmcn1 function^6^, the hypomorphic phenotype in *fndc3a*^*wue1/wue1*^ mutants is milder and locally restricted corresponding to its more specific expression pattern. One cellular consequence of Fndc3a reduction was disturbance of the actinotrichia and collagen fibers assembly process during development, as indicated by altered Col2a location in *fndc3a*^*wue1/wue1*^ mutants and by EM imaging. Collagen helix assembly is performed at the ER and is controlled by chaperones, especially Hsp47/SerpinH1^46^. Interestingly knockdown of *hsp47*/*serpinH1* results in a phenotype resembling *fndc3a*^*wue1/wue1*^ i.e. actinotrichia organization failure and deformations of regenerating fin^47^. In comparison to published loss-of-function mutations in zebrafish collagen genes (e.g. *col1a1*: chi, *Chihuahua; col9a1: prp, persistent plexus*) which result in characteristic, severe bone growth defects, variable skeletal dysplasia and vascular plexus formation^48,49^, the *fndc3a*^*wue1/wue1*^ mutation has only a mild effect. Interference also with expression domains of mesenchymal markers, e.g. *myoD*, suggests that Fncd3a is required for the establishment of ventral cell fates of the developing median fin fold, potentially by setting up the correct cellular structure in this region.

The process of median fin fold development is tightly regulated by modulation of epidermal cell shape and correct ECM assembly^8,9^. TP63 positive epidermal cells are still present in the *fndc3a*^*wue1/wue1*^ mutants during median fin fold development 20 to 48 hpf, but show reduced numbers at ventral positions, delayed ventral fin fold growth and altered morphological properties. Fndc3a shares fibronectin domain III protein domains with other well studied factors like Fibronectin 1, which are known to interact with prominent ECM proteins^50^ and are essential for matrix assembly^51^. The observed breakup of correct epidermal cell assembly and appearance of cavities in the ECM of basal epidermal cells after reduction of Fndc3a function indicate that Fndc3a might play a similar role in correct ECM assembly and establishment of correct cellular structures in the median fin fold. Potential downstream effects like misplaced mesodermal cells, loss of actinotrichia fibers and detached epidermal cells are most likely explained by deranged ECM structure in the early epidermal cell layer after Fndc3a reduction. Mesenchymal cells for example migrate after their induction along predetermined ECM structures and actinotrichia fibers to form the fin skeleton^33^. Irregular skeletal formation of caudal structures, thus, might be a consequence of early developmental irregularities. Future experiments will have to clarify if binding of Fndc3a to integrins is abandoned in *fndc3a*^*wue1/wue1*^ mutants, thereby directly influencing cell adhesion, basal epithelium establishment, and signaling^52,53^. This idea is supported by the similarity of *fndc3a*^*wue1/wue1*^ fin fold defects to laminin (*lmna5)* and integrin (*itga3*) mutants^6,9,21^ and the occurrence of aberrant cells in the epidermal layer of regenerates in the *fndc3a*^*wue1/wue1*^ mutants. Moreover, our experiments do not rule out a potential intracellular function of Fndc3a. Protein localization by Carrouel *et al*. (2008) clearly detected FNDC3A in the Golgi apparatus of human odontoblast. Reduction of Fndc3a function therefore may also result in hampered protein processing within this organelle and thereby interfere with modification of ECM proteins or membranous export.

**Figure 7:**
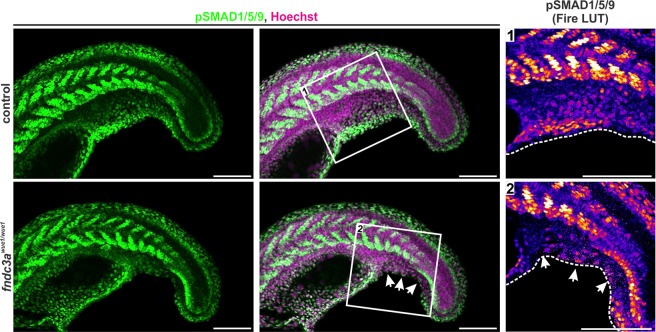
Investigation of TGF-beta/BMP signals in the median fin fold of *fndc3a*^*wue1/wue1*^ mutants. Images show tail structures of representative control and *fndc3a*^*wue1/wue1*^ mutant 22 hpf embryos stained for phosphorylated SMAD (pSMAD1/5/9) by immunofluorescence. Lack of pSMAD positive cells is observed in ventral median fin fold structures in *fndc3a*^*wue1/wue1*^ mutants. All pictures display maximum intensity projections. White arrows indicate loss of pSMAD signals. Fire LUT shows pseudo-colored pSMAD1/5/9 signals. Dashed white lines indicate median fin fold borders. Scale bars: 50 µm.

The severe tail phenotype observed in a minority of adult *fndc3a*^*wue1/wue1*^ fish and in transient double CRISPR injected individuals (Fig. S4) suggests a stronger phenotype after complete loss of Fndc3a and points towards interference with prominent signaling pathways, e.g. BMP and/or TGF-beta signaling. Especially the phenotypic similarity to *smad5* (*somitabun* or *piggytail)*^54,55^, *tll1* (*mini fin* or *tolloid*)^7,56,57^ and *bmp1a* (*frilly fins*)^35^ zebrafish mutants imply an interplay between Fndc3a function and these signaling pathways. The activation of serine/threonine kinase receptors of the TGF-beta superfamily results in phosphorylation of pathway specific intracellular R-SMAD proteins, i.e. SMAD1/5/9, and thereby executing their transcriptional function^58^. Initial experiments in *fndc3a*^*wue1/wue1*^ mutants show that at 22 hpf active TGF-beta/BMP signals as essential patterning factors are lost at ventral median fin fold positions (Fig. 7). This observation is in accordance with investigations focusing on Fndc3b/fad104, which state direct protein-protein interaction of Fndc3b to SMAD proteins and the ability for Fndc3b to act as regulator of BMP/SMAD signaling during calvarial ossification in mice^59,60^. At the moment a similar interaction for Fndc3a and SMAD proteins has not been reported, but our experiments strongly indicate a connection of the *fndc3a*^*wue1/wue1*^ phenotype to loss of SMAD transmitted BMP signals in zebrafish.

Besides median fin fold development we investigated a potential function of Fndc3a during caudal fin regeneration. In accordance with median fin fold development, regeneration and blastema formation depends on correct epidermal cell assembly and a distinct ECM structure^61,62^. We initially detected *fndc3a* transcripts and Fndc3a protein in the distal blastema and in epidermal cell layers of regenerates 2 to 8 dpa. In *fndc3a*^*wue1/wue1*^ mutants we observed several temporal effects on caudal fin regeneration i.e. detached epidermal cells, Col2a accumulation, disorganized epidermal cells layers and lack of actinotrichia. These are comparable to the observed developmental phenotype within the median fin fold and indicate ECM malformation to be causative for this effect on adult structures as well. Our regeneration experiments hint to rather minor and temporal effects of Fndc3a on regeneration during initiation and the first few days of regeneration.

**Figure 8:**
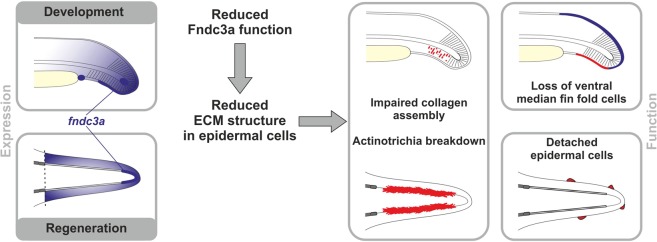
Model of *fndc3a* function during zebrafish median fin fold development and caudal fin regeneration. Expression and localization of *fndc3a* was detected during early phases of median fin fold development and in caudal fin regenerates. Reduced Fndc3a function resulted in prominent changes of ECM structure in epidermal cells. During development this results in impaired collagen assembly of actinotrichia fibers and loss of ventral median fin fold cells. During regeneration altered ECM structure results in actinotrichia breakdown and detached epidermal cells.

In summary, investigation of *fndc3a* expression and function in zebrafish reveals a transient and spatially restricted role of this genetic factor during extremity development and during fin regeneration (Fig. 8). The observed effects after reduction of Fndc3a function on actinotrichia fibers and correct fin morphology are probably secondary and provoked by cellular changes. Fndc3a can influence TP63 positive epidermal cells by altering cell shape or cell adhesion during extremity development. Most likely disruption of correct ECM structure in basal epidermal cells is the consequential underlying cellular mechanism responsible for the observed fin malformations and signaling changes. Our results therefore demonstrate a cellular link between median fin fold development and caudal fin regeneration due to the necessity of correct cell shape and tissue cohesion in both processes via Fndc3a.

## Materials and Methods

### Animal maintenance

Laboratory zebrafish embryos (*Danio rerio*) of the *AB/TU and AB/AB* strain (ZDB-GENO-010924-10; ZDB-GENO-960809-7) were maintained as described by Westerfield^63^ under standard aquatic conditions at an average of 24 °C water temperature. Embryos were staged by morphological characteristics according to Kimmel *et al*.^64^ hpf indicate hours-post fertilization at 28.5 °C. All procedures involving experimental animals were performed in compliance with German animal welfare laws, guidelines, and policies. Generation of *fndc3a*^*wue1/wue1*^ mutants and fin clipping was approved by the Committee on the Ethics of Animal Experiments of the University of Würzburg and the “Regierung von Unterfranken” (Permit Number: DMS-2532-2-13 and DMS-2532-2-9). The generated *fndc3a*^*wue1/wue1*^ line was submitted to ZFIN.org (ZFIN ID: ZDB-ALT-170417-3).

### CRISPR/Cas9 system and construction of short guiding RNA (sgRNA) constructs

For oligo cloning of sgRNA target sequences the previously published pDR274 vector was used^27^ (Addgene Plasmid #42250; sequences of primers used in this study are given in Table S1). For *cas9* RNA synthesis the MLM3613^27^ (Addgene Plasmid #42251) or the pCS2-nCas9n vector^28^ (Addgene Plasmid #47929) were utilized. Both vectors were purchased from Addgene (www.addgene.org; Cambridge, USA). For designing and constructing of sgRNAs the open access ZiFit Targeter software (http://zifit.partners.org/ZiFiT/) was used. Specific target sites were identified by alignments of zebrafish and human sequences. sgRNA target site (GGATTCCAGGCCAGTTATGA) is located in exon 13 of *fndc3a* (ENSEMBL Zv9 Transcript: ENSDART00000097261) and targets the second Fibronectin type III domain, while sgRNA target site in exon 18 (GGCGTACAGTGGTTCGGCTC) targets the third Fibronectin type III domain.

### sgRNA transcription and microinjection

sgRNAs were transcribed via the MAXIscript T7 kit (Ambion/ life technologies, Darmstadt, Germany) and were puridied via phenol/chloroform extraction. *Cas9* RNA was transcribed via the mMESSAGE mMACHINE kit (Ambion/ Life Technologies, Darmstadt, Germany) and subsequently cleaned via RNeasy purification kit (Qiagen, Venlo, Netherlands). If the MLM3613 vector was used for Cas9 Synthesis, polyA tail synthesis was performed with *E.coli* Poly(A) Polymerase (New England Biolabs, Ipswich, MA, USA) prior to purification. One cell stage zebrafish embryos were injected with solutions comprising sgRNA (25–50 ng/µl each), *cas9* RNA (75–100 ng/µl), Phenol red (pH7.0; 0.05% final concentration; for visualization of injection solution) and Fluorescein isothiocyanate-dextran (Sigma-Aldrich; 1 mg/µl). Positively injected embryos were identified 24 hpf by transient green fluorescence of Fluorescein isothiocyanate-dextran and were raised for line establishment.

### Whole mount RNA ***in-situ*** hybridization

RNA *in situ* hybridization was performed according to standard protocols^65,66^. Proteinase K incubation and NBT/BCIP staining times were adjusted to age of embryos and to investigated tissues. RNA probes were synthesized from cloned partial mRNA sequences of target genes using the DIG or FLU RNA Labeling Kit (Roche, Basel, Switzerland). We used a *fndc3a* cDNA fragment of 595 bp size (used primers: zf_fndc3a_ribo_fwd2 and zf_fndc3a_ribo_rev2) to synthesize a specific anti-sense RNA probe. Sense probes were synthesized as negative control for each anti-sense probe and were used under the same reaction conditions. Primers used for probe cloning are listed in Table S1.

### Immunofluorescence and histology

Immunofluorescence was performed on embryos, sections or on whole regenerating fins according to standard protocols^16,67^. Primary antibodies used: Fndc3a (HPA008927; Sigma-Aldrich; dilution 1:50–100; Antibody Registry: AB_1078899), Col2a (II-II6B3; anti-mouse; The Developmental Studies Hybridoma Bank; dilution 1:500; Antibody Registry: AB_528165), β-catenin (610153; BD Biosciences; dilution 1:250; Antibody Registry: AB_397554), TP63 (ab735; Abcam; dilution 1:200; Antibody Registry: AB_305870), Phospho-Smad1 (Ser463/465)/Smad5 (Ser463/465)/Smad9 (Ser465/467) clone D5B10 (13820; anti-rabbit; Cell Signaling Technology; 1:200 dilution; Antibody Registry: AB_2493181). Except Fndc3a, all used antibodies have been used previously for experiments in zebrafish and validated protocols have been previously published. Specificity of the used Fndc3a antibody was validated by the Human Protein Atlas project for usage in human tissues (https://www.proteinatlas.org/ENSG00000102531-FNDC3A/antibody). Validation for usage in zebrafish was performed by epitope sequence analysis, antibody dilution series, and incorporation of adequate negative controls in all experiments. Cell nuclei were counterstained with Hoechst 33258 (Sigma-Aldrich; dilution 1:5000). F-Actin was stained with Acti-stain 488 phalloidin (Cytoskeleton, Inc.; PHDG1-A; dilution 1:20). Whole mount alcian blue staining was performed according to Walker and Kimmel^68^. H&E staining on 10 µm paraffin sections was performed according to a general histology protocol using Mayer’s Hematoxylin and Eosin.

### Fin regeneration experiments

For fin amputation experiments adult zebrafish (mixed sex, age 3–5 months, 3 independent experiments) were anesthetized with tricaine (MS-222; 3-amino benzoic acid ethyl ester; final concentration ~150 mg/l) prior to amputation of ~20% length the of caudal fin. Images were taken 0, 2, 6, 8 and 10 days post-amputation (dpa) under a stereomicroscope. To increase the expected phenotype fish were kept at an increased temperature (32 °C) during the experiments. Regenerate samples for immunofluorescence, *in-situ* hybridization, genotyping, or histology were taken by a second amputation anterior to the first lesion site (2, 4, 6 and 8 dpa).

### Image acquisition and quantification

Images were acquired depending on the experiment either with a Leica S8 APO Stereomicroscope (whole embryos), a Zeiss Imager A1 (*in-situ* hybridizations) or a Nikon A1+ Laser scanning confocal microscope (Immunofluorescence). Image acquisition was performed via device specific cameras/detector and corresponding software (Leica Application suite; Zeiss AxioVision; Nikon NIS-Elements). Further image analyses and quantifications were performed with ImageJ/Fiji (https://fiji.sc/). For figure arrangement CorelDraw Graphics Suite x7 software (Corel Corporation) was used.

Quantification of median fin fold width was performed with ImageJ/Fiji by length measurement of maximum intensity projections of TP63/Hoechst stained embryos 24 and 48 hpf. Each embryo was measured at three independent positions at the dorsal or ventral median fin fold. Median fin fold width was measured between the apical fin and somite borders. Quantification of epidermal cells in 24 hpf embryos was performed by automated counting of TP63 positive cells in confocal stacks (size z: 40; z-step: 2 µm) on the complete ventral and dorsal median fin folds. While in 48 hpf embryos three independent positions of the same size (ventral and dorsal median fin fold, tail tip; 8800 µm^2^) were investigated per sample. Used ImageJ plugins and commands: *Stack/Z Project*, *Despeckle*, *Watersheed*, *Analyze particles* (size 2–10 µm, circularity 0.5–1.0). A minimum of 12 samples for each condition was investigated.

### Electron microscopy

Embryos or regenerates were washed with PBS and fixed (2.5% glutaraldehyde, 50 mM cacodylat pH 7.2, 50 mM KCl, and 2.5 mM MgCl_2_) overnight at 4 °C. Subsequently, embryos or tissues were washed five times with 50 mM cacodyl buffer (pH 7.2) and fixed for 4 h with 2% OsO_4_ in 50 mM cacodylat (pH 7.2) buffer. Embryos or tissues were subsequently stained with 2% uranylacetate overnight. After gradual dehydration with ethanol, they were transferred to propylenoxid and embedded in Epon (SERVA Electrophoresis GmbH, Heidelberg, Germany). Ultrathin sections were analyzed using an EM10 from Zeiss (Oberkochen, Germany).

### qPCR experiments

For qPCR experiments RNA was extracted from pools of 12 embryos each (age 24 hpf; F3 *fndc3a*^*wue1/*^^+^ and *fndc3a*^*wue1/wue1*^ generation; control: *AB*). For each genotype three independent pools were generated and compared. For cDNA synthesis 1 µg RNA was transcribed into cDNA and further analyzed in a ViiA7 Real-Time PCR System (Thermo Fisher Scientific). Each group and primer sample was analyzed in triplicates on a single qPCR plate utilizing HOT FIREPol Eva Green Mix Plus (Solis BioDyne). Data analysis of was performed via QuantStudio Real-Time PCS Software v1.1 by ΔΔCt method. *AB* control samples were used as reference sample for relative comparison. Amplification of *gapdh* and *eef1a1l1* was used as endogenous controls. Used primer pairs, amplicon sizes and targeted regions are noted in Table S1.

## Supplementary information

Supplementary Data 1
