# Supplementary material for: ECM alterations in Fndc3a (Fibronectin Domain Containing Protein 3A) deficient zebrafish cause temporal fin development and regeneration defects
Source: Sci Rep. 2019 Sep 16;9:13383. doi: 10.1038/s41598-019-50055-w (PMC6746793; doi:10.1038/s41598-019-50055-w)
Supplement: Supplementary file 1 — Supplementary Data 1 [file 41598_2019_50055_MOESM1_ESM.docx]

**Supplementary information**

**ECM alterations in Fndc3a (Fibronectin Domain Containing Protein 3A) deficient zebrafish cause temporal fin development and regeneration defects.**

Daniel Liedtke^*^, Melanie Orth, Michelle Meissler, Sinje Geuer, Sabine Knaup, Isabell Köblitz, Eva Klopocki

**Table S1:** Primers used in this study.

| Name | Primer sequence | amplicon size [bp] | purpose |
| --- | --- | --- | --- |
| CRISPR-zf-fndc3a-fwd01 | TAGGATTCCAGGCCAGTTATGA |  | sgRNA cloning, targeting exon 13 |
| CRISPR-zf-fndc3a-rev01 | AAACTCATAACTGGCCTGGAAT |  | sgRNA cloning, targeting exon 13 |
| fndc3a_CRISPR2_fwd | TAGGCGTACAGTGGTTCGGCTC |  | sgRNA cloning, targeting exon 18 |
| fndc3a_CRISPR2_rev | AAACGAGCCGAACCACTGTACG |  | sgRNA cloning, targeting exon 18 |
| zf_fndc3_gDNA_fwd | cttggcatccatcaatcgta | 889 | gDNA amplification and sequencing |
| zf_fndc3_gDNA_rev | gcaaaagtggattcggacat | 889 | gDNA amplification and sequencing |
| zf_fndc3_gDNA_fwd2 | gtgcgttcgtgtgtgtttct | 530 | gDNA amplification and sequencing |
| zf_fndc3_gDNA_rev2 | acagtcctccacctcctcct | 530 | gDNA amplification and sequencing |
| Fndc3a_CRISPR1-OFF01-fwd | CGCTTTCCTGTACAAAGTCCA | 336 | gDNA amplification and sequencing; offsite target 1  chr11:+5647495 |
| Fndc3a_CRISPR1-OFF01-rev | gagagatttgttgggggtga | 336 | gDNA amplification and sequencing; offsite target 1  chr11:+5647495 |
| Fndc3a_CRISPR1-OFF02-fwd | TGCATTCAAATATTTTACCCATTT | 328 | gDNA amplification and sequencing; offsite target 2 chr15:-21353577 |
| Fndc3a_CRISPR1-OFF02-rev | TGGCTTAAGTTAATGCTGTCTGG | 328 | gDNA amplification and sequencing; offsite target 2 chr15:-21353577 |
| Fndc3a_CRISPR1-OFF03-fwd | AAATGTACCGATCGCCTCTG | 365 | gDNA amplification and sequencing; offsite target 3  chr4:-36523722 |
| Fndc3a_CRISPR1-OFF03-rev | TTTACGCATTACGCATCAGC | 365 | gDNA amplification and sequencing; offsite target 3  chr4:-36523722 |
| zf-fndc3a-fwd1 | TCCCCCAGTACATCTCCCAG | 740 | Cloning and riboprobe synthesis |
| zf-fndc3a-rev1 | ACAGCTCAGAGTGGTGAAGC | 740 | Cloning and riboprobe synthesis |
| zf_fndc3a_ribo_fwd2 | AGGTGATGATGAGCCCAAAG | 594 | Cloning and riboprobe synthesis |
| zf_fndc3a_ribo_rev2 | CGAATGCATAGCCATGTGAC | 594 | Cloning and riboprobe synthesis |
| zf_fndc3a_fwd1 (+Sp6) | GATTTAGGTGACACTATAGAAGC TCCCCCAGTACATCTCCCAG |  | riboprobe synthesis |
| zf_fndc3a_rev1 (+T7) | GTAATACGACTCACTATAGGGCG ACAGCTCAGAGTGGTGAAGC |  | riboprobe synthesis |
| Zf_fndc3a_splMO_ex9_fwd | AGGCTGTGAGCTTCACCACT | 382 | Morpholino test |
| Zf_fndc3a_splMO_ex12_rev | CGAATGCATAGCCATGTGAC | 382 | Morpholino test |
| Zf_fndc3a_splMO_ex11_fwd | AACGGATGCTACGTTTGTCC | 418 | Morpholino test |
| Zf_fndc3a_splMO_in11-12_rev | tcatattagcactcgactaacagc | 418 | Morpholino test |
| zf_fndc3a_qPCR1_fwd1 | AGGCTGTGAGCTTCACCACT | 178 | qPCR, intron-spanning from exon 9 to 11, before mutation site (GRCz9: ENSDARG00000067569) |
| zf_fndc3a_qPCR1_rev1 | ACAAACGTAGCATCCGTTCC | 178 | qPCR, intron-spanning from exon 9 to 11, before mutation site  (GRCz9: ENSDARG00000067569) |
| zf_fndc3a_qPCR2_fwd1 | CAGCGTTTACACCTTCACCA | 153 | qPCR, intron-spanning from exon 27 to 28, after mutation site (GRCz9: ENSDARG00000067569) |
| zf_fndc3a_qPCR2_rev1 | TGCATGCATTGCAAACAGTA | 153 | qPCR, intron-spanning from exon 27 to 28, after mutation site (GRCz9: ENSDARG00000067569) |
| Zf_gapdh_qPCR_fwd1 | GTGGAGTCTACTGGTGTCTTC | 173 | qPCR, intron spanning from exon 5 to 7; housekeeping reference, gapdh (GRCz10: ENSDART00000063800) |
| ZF_gapdh_qPCR_rev1 | GTGCAGGAGGCATTGCTTACA | 173 | qPCR, intron spanning from exon 5 to 7; housekeeping reference, gapdh (GRCz10: ENSDART00000063800) |
| ef1a1-f01 | GCCCCTGGACACAGAGACTTCATCA | 211 | qPCR, intron spanning from exon 3 to 4; housekeeping reference, eef1a1l2 (GRCz10: ENSDARG00000020850) |
| ef1a1-r01 | AAGGGGGCTCGGTGGAGTCCAT | 211 | qPCR, intron spanning from exon 3 to 4; housekeeping reference, eef1a1l2 (GRCz10: ENSDARG00000020850) |

Sequences of primers used for cloning of other in-situ probes are available on request.

**Supplementary methods**

**CRISPR validation and line establishment.**
Functionality of used sgRNAs and Cas9 RNA was assessed by sequencing gDNA of eight injected embryos 4-5dpf for locus specific alterations. sgRNA/Cas9 RNA injected embryos were raised until adulthood for next generation crossings. Sequencing of eight F1 siblings per crossing indicated a number of founder crosses. One fish line with a 5bp substitution at the targeted locus was used for further experiments. Heterozygous founder fish were identified by fin-clipping and genotyping. Incrossing of F1 individuals resulted in a *fndc3a^wue1/wue1^* homozygous mutant line and were identified by gDNA sequencing. Potential off-site targets of the used sgRNA were identified by the corresponding software, e.g. CCtop [^62^](#_ENREF_62), ZiFit Targeter and the Zhang lab CRISPR design tool. Targets with highest scores were sequenced in *fndc3a^wue1/wue1^* mutants to validate a normal genotype at these loci (Fig. S1). The institute specific line designation for the generated line is *fndc3a^wue1^* and has been submitted to zfin.org (http://zfin.org/ZDB-ALT-170417-3).

**Genomic DNA extraction and sequencing.**Extraction of genomic DNA from fin clips or from whole 4-5dpf old embryos was performed by Proteinase K digestion as previously described [^63^](#_ENREF_63). 2µl of eluted genomic DNA (approx. 50ng/µl) was used for PCR amplification, with subsequent clean-up and Sanger sequencing. Sequences of primers used in this study are given in Table S1. Sequencing results were analyzed with “ApE” (<http://biologylabs.utah.edu/jorgensen/wayned/ape/>) and “CodonCode Aligner” (CodonCode Corporation, Centerville, MA, USA) software packages.

**Morpholino and RNA injections**
Knockdown experiments were performed by microinjection of *fndc3a* splice Morpholino (5´ GCGTTCTGAGCAATACACACCTGAC-3´; Gene Tools, LLC,USA) of different concentrations into 1 cell-stage embryos. The Morpholino was targeted against the splice donor site of *fndc3a* exon 11. Analysis of Morpholino function was performed by RT-PCR via detection of larger, incorrectly spliced mRNA products or increased detection of intron specific fragments (exon 9 to 12 spanning primers: 387bp for correctly spliced product, 2070bp for incorrectly spliced product with stabilized intron; exon 11 to intron 11-12 primers: 418bp product indicates presence of intronic sequence on cDNA level). Stabilization of intron 11-12 results after 60bp in a number of premature Stop codons in the coding sequence.
RNA rescue or overexpression experiments were performed by microinjection of capped, full length human FNDC3A RNA into 1 cell-stage embryos (CDS originating from Clone ID: 9052821; GE Dharmacon). Human FNDC3A was used, as the complete coding sequence of the zebrafish *fndc3a* gene had not correctly been annotated in the genomic databases of that time. A comparison between the complete human FNDC3A and the lagest zebrafish *fndc3a* fragment (Zv9 ENSDART00000097261, missing approx. 60 amino acids at the N-terminus) indicates 65.2% similar and 49.8% identical amino acids.

**Supplementary figures**


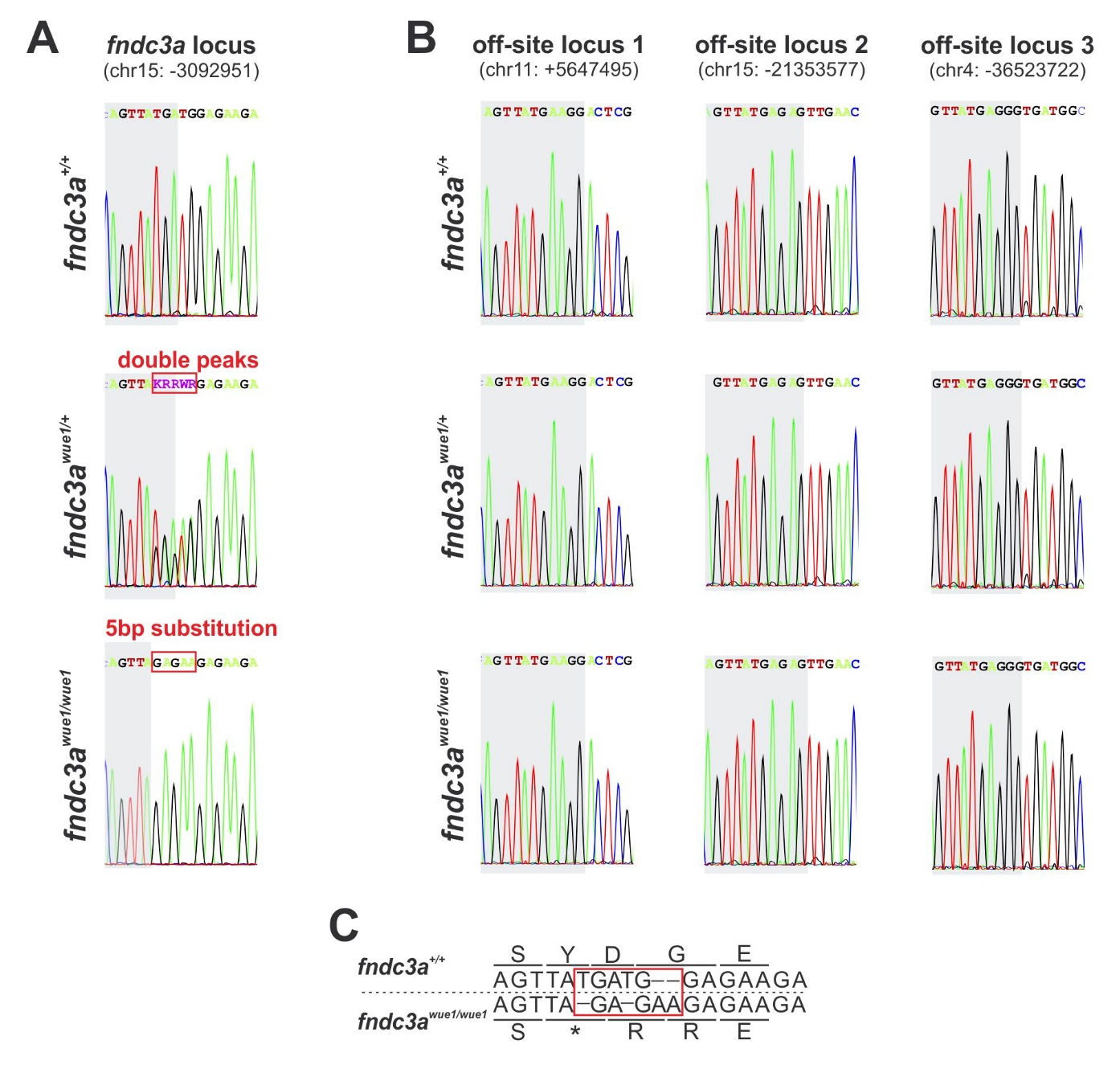


**Fig. S1: Validation of specific mutations and off-site target analyses in *fndc3a* mutants.**

Representative sequencing chromatograms for the *fndc3a* locus (A) and three off-site targets with highest predicted cutting probability (B) are shown. The generated *fndc3a* line carries a 5bp substitution at the expected position, while the other investigated loci show no sequence alterations. (C) cDNA sequence alignments between controls and *fndc3a^wue1/wue1^* mutants showed nucleotide and amino-acid sequence alterations, resulting in a premature stop codon. sgRNA or potential off-site target sequences are marked in grey.


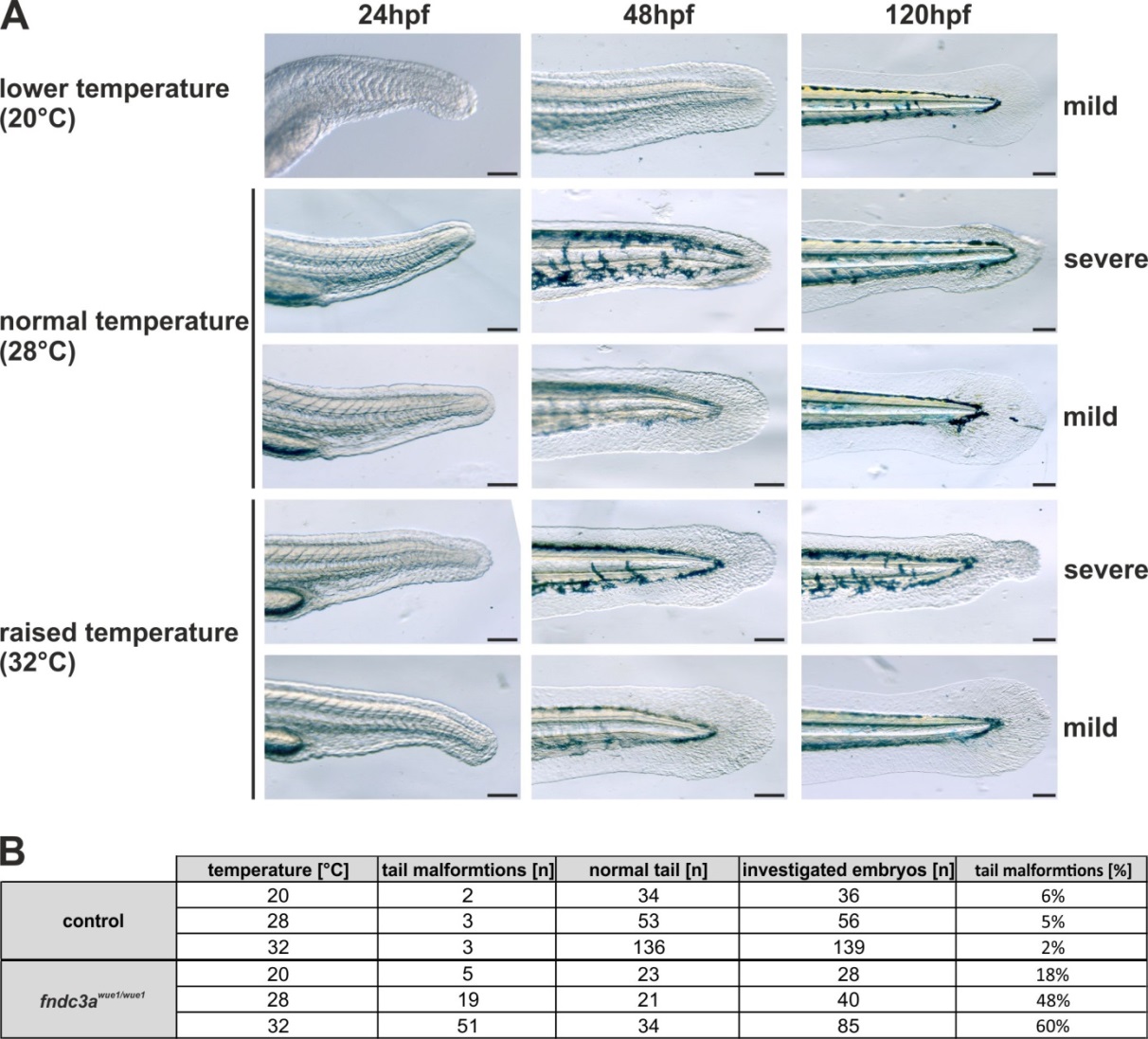


**Fig. S2: Temperature dependency of *fndc3a^wue1/wue1^* fin phenotype during fin development**
*fndc3a^wue1/wue1^* embryos of a single batch were separated into three groups and raised at different temperatures. (A) Representative phenotypes with a mild or severe appearance, respectively. (B) Quantification of tail phenotypes after incubation at different temperatures.

Scale bars: 100µm.

**
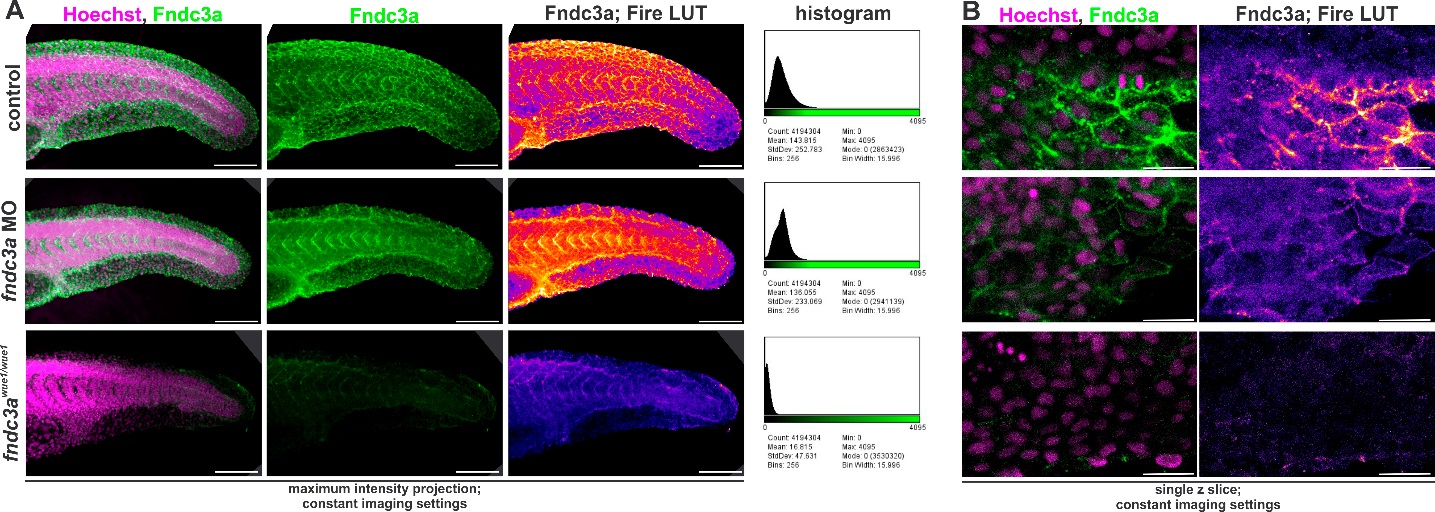
**

**Fig. S3: Investigation of Fndc3a protein loss in morphants (MO) and *fndc3a^wue1/wue1^* mutants 22hpf.**
(A) Images show tail structures of representative embryos stained for Fndc3a and imaged under constant settings. Histograms show fluorescence intensity quantification of GFP channel pixels. (B) High resolution images of single z slices focusing on ventral median fin fold structures.
Scale bars in A: 50µm, in B: 20µm.


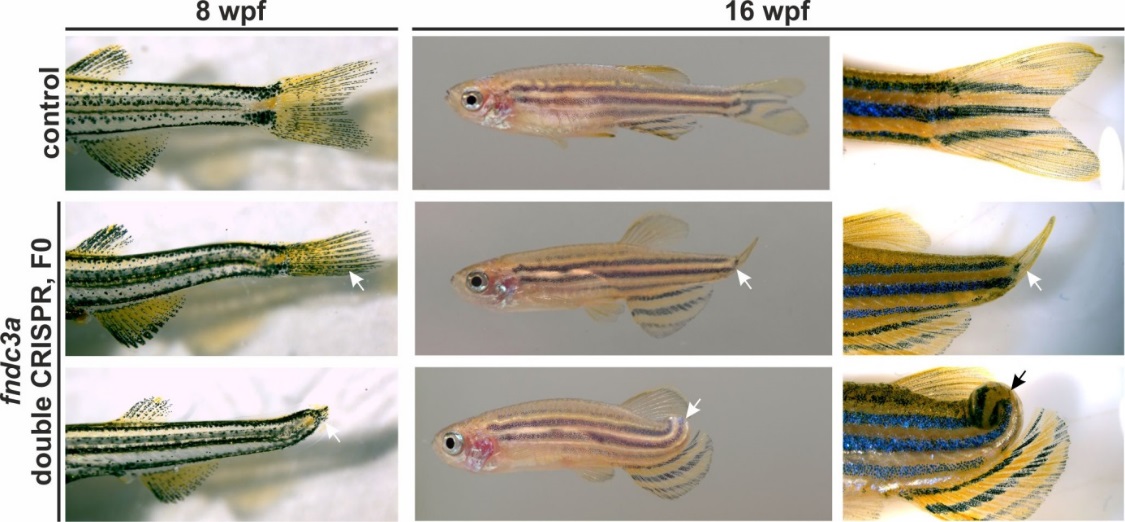


**Fig. S4: Phenotypic effect of transient *fndc3a* double CRISPR injections.**Different F0 individuals injected with two *fndc3a* gRNA targeting exon 13 and 18 in the *fndc3a* locus via CRISPR/Cas9 are depicted. A number of these fish showed severe tail malformations 8 and 16 weeks post fertilization (wpf) and indicated a stronger effect than *fndc3a^wue1/wue1^* mutants (n=12/32; arrows indicate tail malformations).


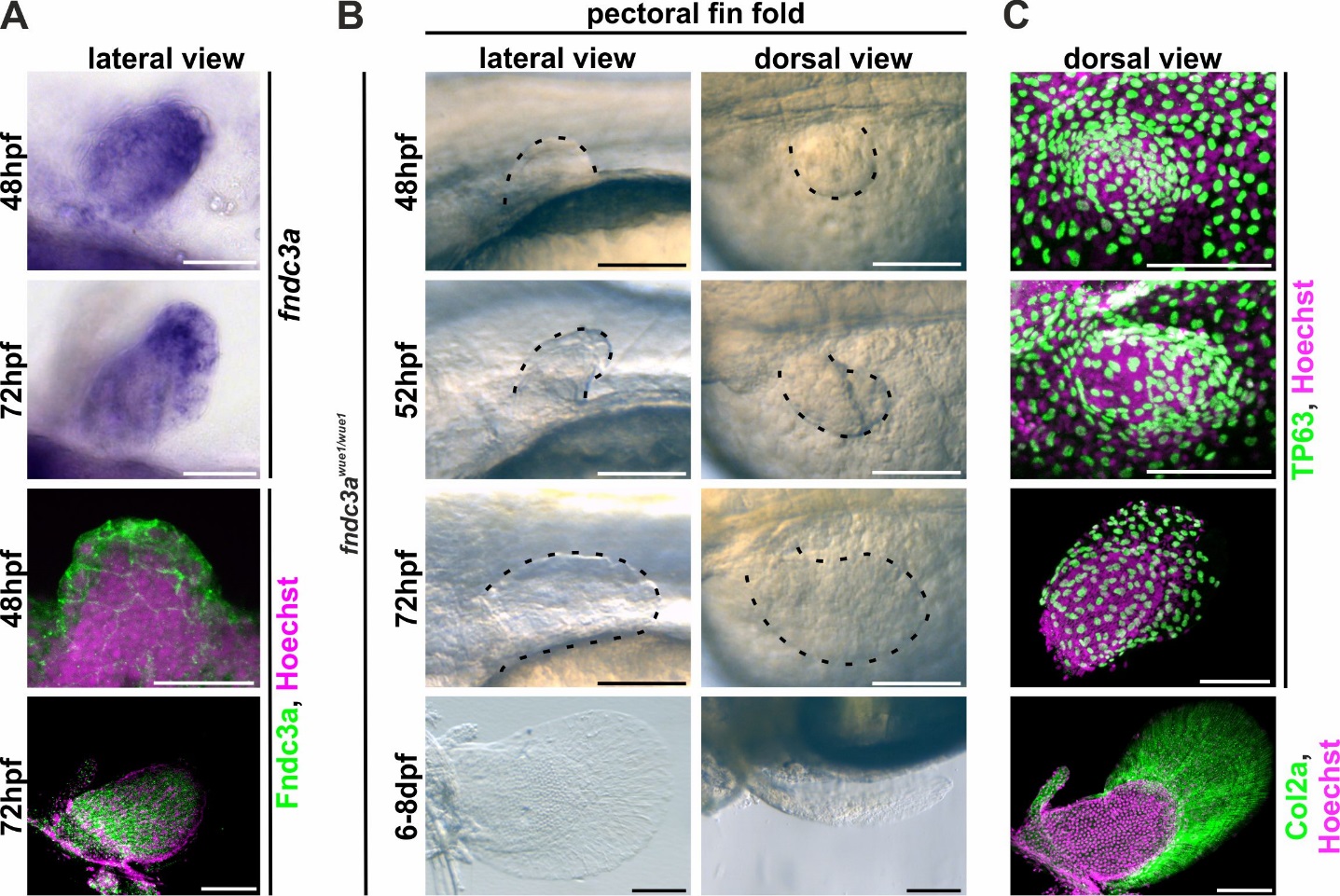


**Fig. S5: Expression of *fndc3a* in pectoral fins and pectoral fin development in *fndc3a^wue1/wue1^*mutants.**

(A) Detection of *fndc3a* mRNA transcripts in pectoral fins 48 and 72 hpf by in-situ hybridization and localization of Fndc3a protein by immunofluorescence at the same stages. (B) Phenotypic investigation of pectoral (left two rows, showing lateral and dorsal views) and median fin fold development at different stages of development in *fndc3a^wue1/wue1^*mutants. Images show examples of single individuals. (C) Immunofluorescence staining for TP63 and Col2a indicated no changes in pectoral fin development of *fndc3a^wue1/wue1^*mutants.
Dashed lines indicate fin fold areas. Scale bars: 100µm.


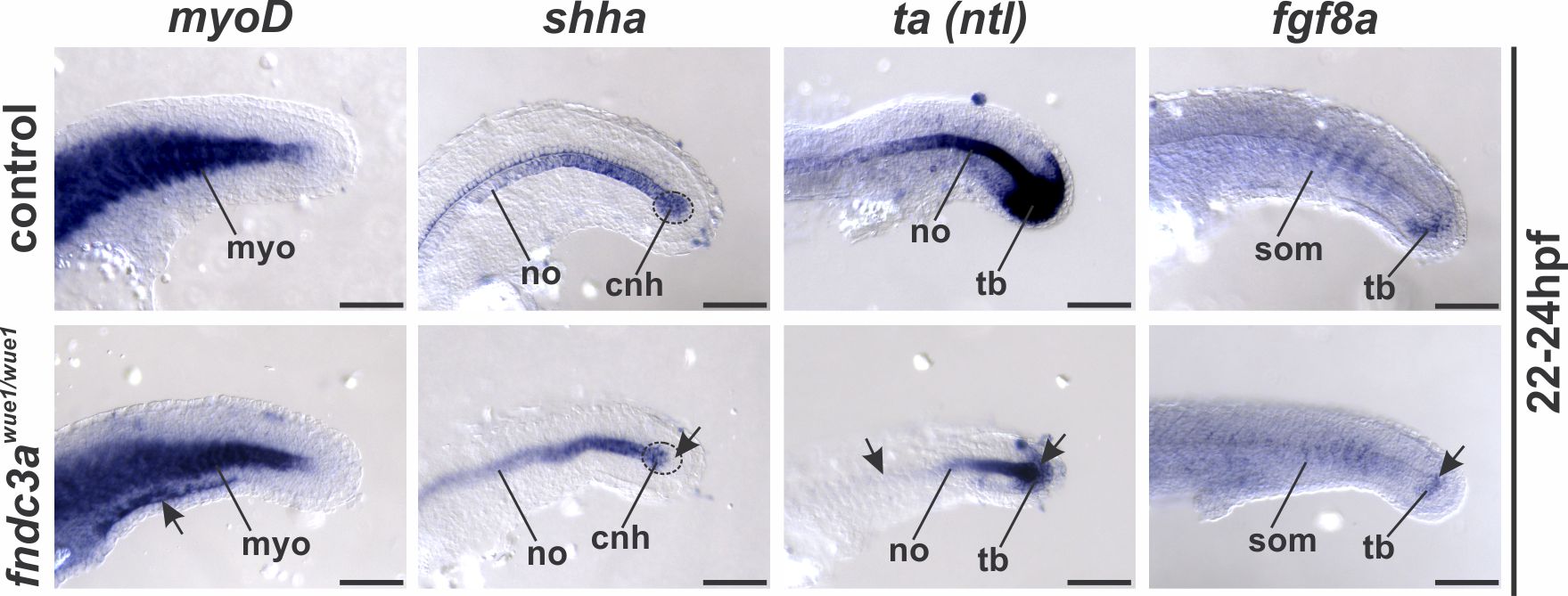


**Fig. S6: Alterations of mesodermal gene expression domains in *fndc3a^wue1/wue1^* mutants.**Gene expression of different mesodermal factors was investigated by in-situ hybridization in *fndc3a^wue1/wue1^* mutants. All investigated genes were still expressed at 22-24hpf, but showed alterations in their expression domains (arrows; *myoD*: 12/22 embryos; *shha*: 7/17 embryos; *ta*: 5/13 embryos; *fgf8a*: 5/13 embryos). Additional *myoD* positive cells could be detected ventrally to the wildtype expression domain in the myotome (myo), while *shha*, *ta* and *fgf8a* showed reduced expression in notochord (no) cells, the chordo neural hinge (cnh) and in the tail bud (tb). No change was detected for *fgf8a* expression in the somites (som).
Dashed lines indicate area of the cnh region in control embryos. Scale bars: 100µm.

**
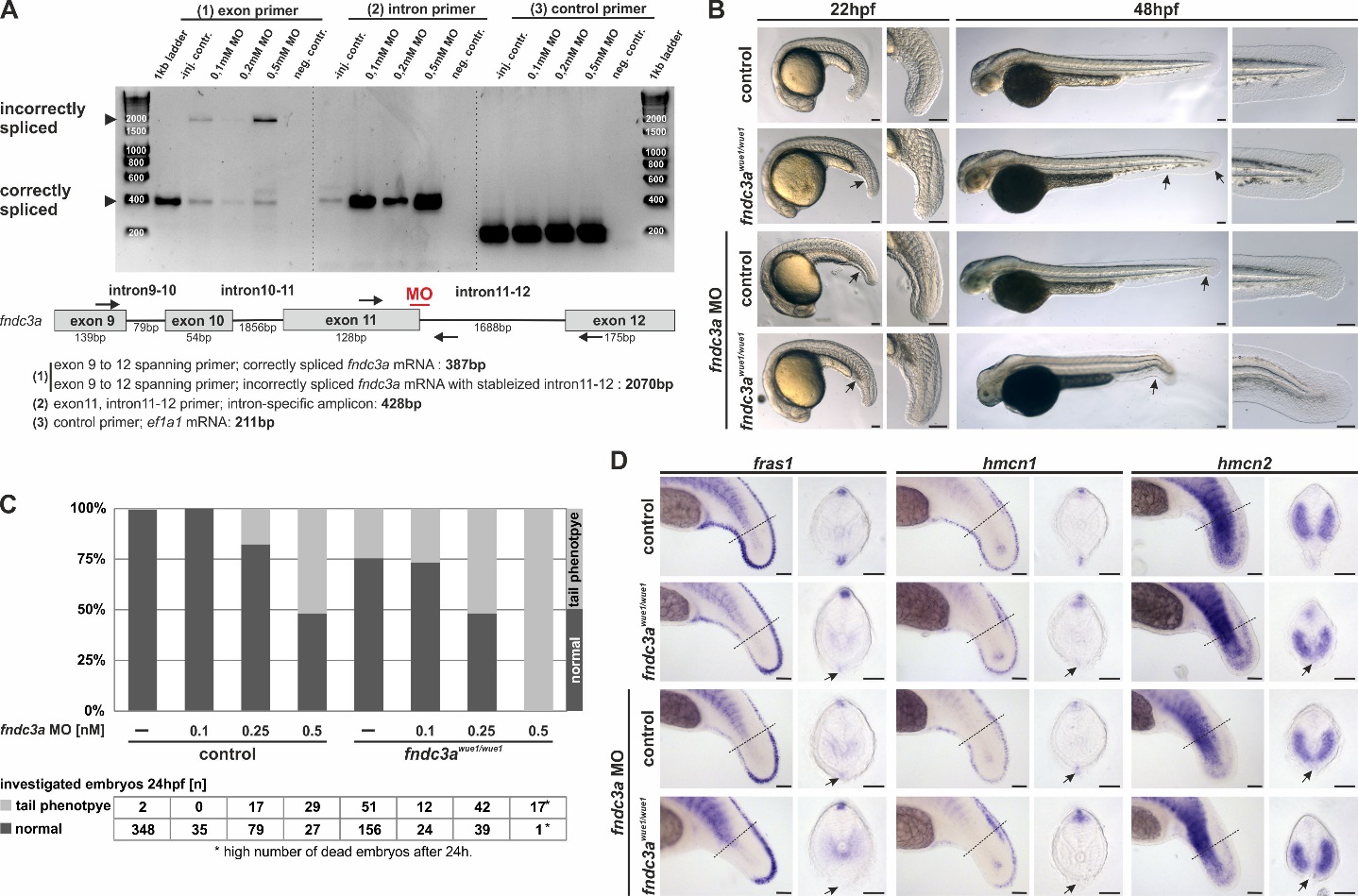
**

**Fig. S7: *fndc3a* splice Morpholino knockdown experiments.**(A) Injection of *fndc3a* splice Morpholinos directed against the splice donor site of exon 11 resulted in the stabilization of intron 11-12. cDNAs of embryos injected with different concentrations of *fndc3a* splice Morpholino were analyzed by RT-PCR using either exon 9 to 12 spanning or intron 11-12 specific primers. Exon spanning primers indicated a correctly spliced (387 bp) and a larger incorrectly spliced PCR product (2070 bp). Furthermore, detection of stronger bands specific for intron 11-12 further confirmed this observation. (B) Injection of *fndc3a* splice Morpholino phenotypically resulted in similar tail phenotypes observed in *fndc3a^wue1/wue1^* mutants 22hpf and 48hpf. (C) Quantification of tail phenotypes after Morpholino injection (24hpf at 28°C; statistical significant differences between control and mutant values were detected by a *X^2^* test = 145,32). (D) In-situ hybridization showed loss of mesenchymal gene expression (*fras1*, *hmcn1* and *hmcn2)* after Morpholino knockdown in 22hpf old embryos (control: 0/57; *fndc3a^wue1/wue1^*: 30/58; *fndc3a* 0.25nM MO injection in *AB*: 14/28; *fndc3a* 0.25nM MO injection in *fndc3a^wue1/wue1^*: 12/12*)*.
Dashed lines in D indicate planes of shown sections. Scale bars in B: 100µm; scale bars in D: 50µm.

**
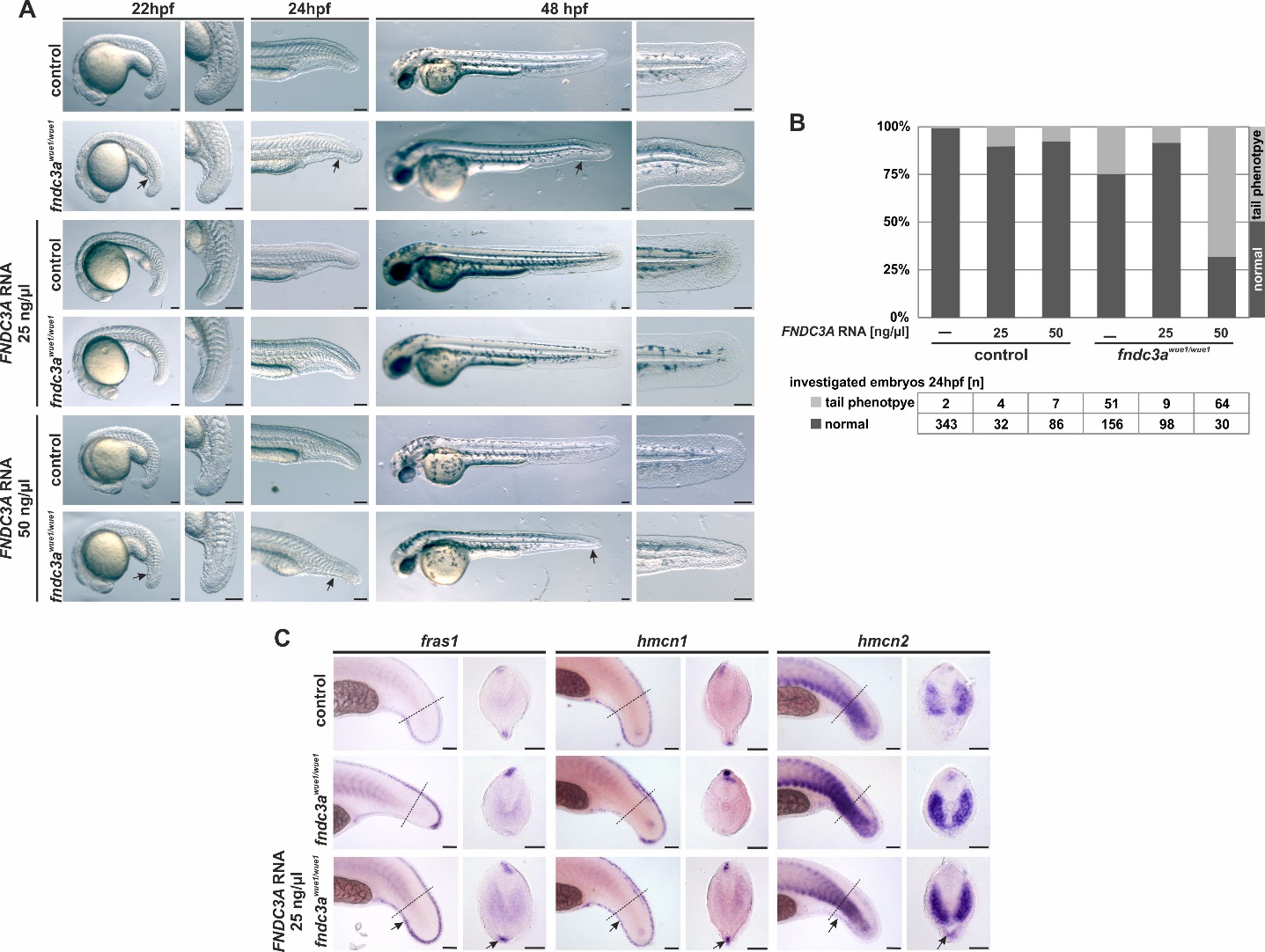
**

**Fig. S8: *FNDC3A* RNA overexpression and mutant rescue experiments.**

(A) Injection of full-length human *FNDC3A* RNA resulted in partial rescue of the *fndc3a^wue1/wue1^* phenotype at lower RNA concentrations (25 ng/µl) and in an overexpression phenotype at higher concentrations (50 ng/µl). (B) Quantification of tail malformations after RNA injection (24hpf at 28°C; statistical significant differences between control and mutant values were detected by a *X^2^* test = 284,44). (C) In-situ hybridization showed regain of ventral median fin fold gene expression; *fras1*, *hmcn1* and *hmcn2*; in *fndc3a^wue1/wue1^* mutants after RNA rescue with 25 ng/µl (control: 0/20; *fndc3a^wue1/wue1^*: 25/55; *fndc3a^wue1/wue1^* RNA injection 25 ng/µl: 4/80; regain of ventral expression is marked with arrows).
Dashed lines in C indicate planes of shown sections. Scale bars in A: 100µm; in C: 50µm.


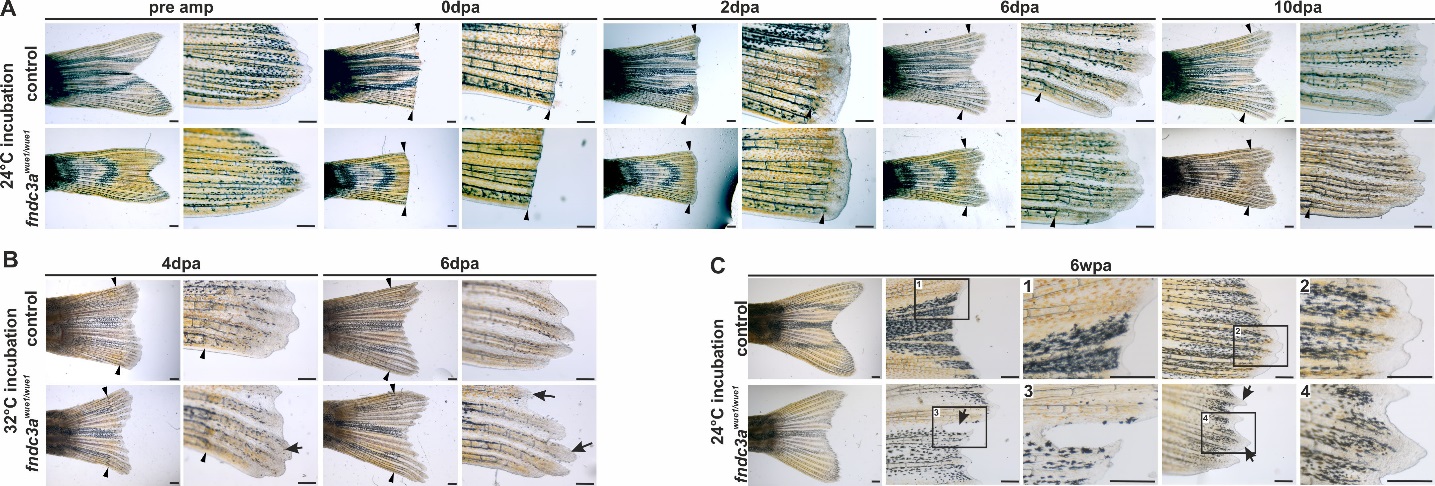


**Fig. S9: Fin regeneration in *fndc3a^wue1/wue1^* mutants.**(A) Regeneration of the caudal fin of in *fndc3a^wue1/wue1^* mutants proceeded with similar speed compared to control fish at 24°C and displayed mild alterations. (B) While, regeneration of the caudal fin of in *fndc3a^wue1/wue1^* mutants at 32°C displayed distinct cellular defects at 4 and 6dpa. (C) Fully regenerated caudal fin 6 weeks after amputation (wpa) showed fin abnormalities, e.g. notches between fin rays.
Arrowheads indicate amputation site; arrows indicate sites of irregular blastema or sites of abnormal fin morphology. Scale bars in A, B and C: 200µm.

**
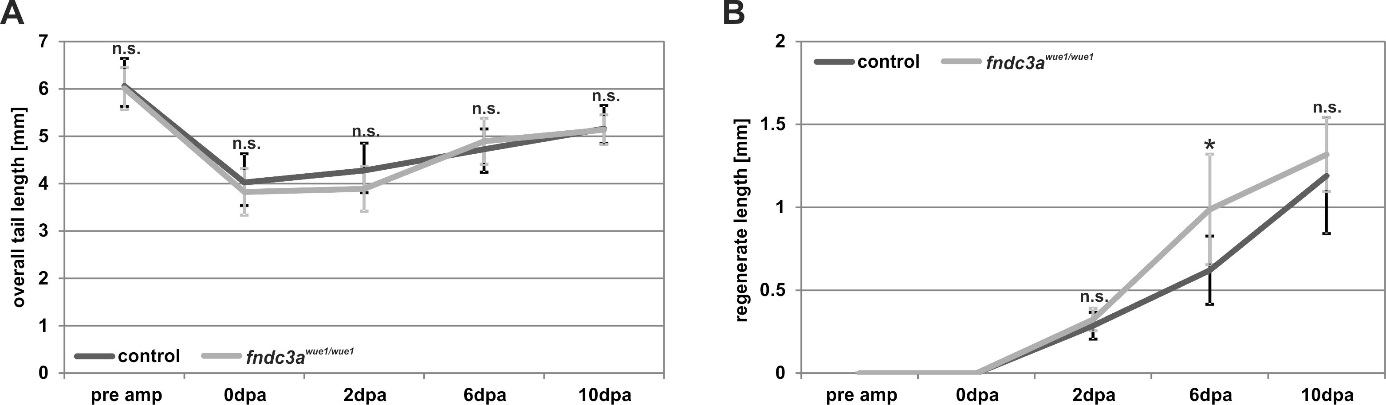
**

**Fig. S10: Quantification of fin length and regenerate length during regeneration in *fndc3a^wue1/wue1^* mutants.**Measurements of overall tail fin length (A) and regenerate length (B) were performed at different time points after amputation. Overall fin length did not show significant differences between control fish and *fndc3a^wue1/wue1^* mutants (n=10 to 22; 24°C; two-tailed Mann-Whitney U test, p<0.05). Regenerate length did not show significant differences between control fish and *fndc3a^wue1/wue1^* mutants 2 and 10dpa, but showed significant differences 6dpa (Mann-Whitney U test = 1.336 x10^-4^; n1=18; n2=22; p<0.05; two-tailed).
